# Controllable molecular generation with fine-tuned flow-matching model

**DOI:** 10.1038/s42004-026-02188-z

**Published:** 2026-09-05

**Authors:** Kunyu Wang, Jon Paul Janet, Alessandro Tibo

**Affiliations:** Molecular AI, Discovery Sciences, R&D, AstraZeneca, Gothenburg, Sweden

**Keywords:** Drug discovery, Cheminformatics, Computational chemistry

## Abstract

Three-dimensional molecular generative models have emerged that produce de novo molecules both unconditionally and conditionally, e.g., within protein pockets. However, steering those models in a specific region of the chemical space that satisfies a set of desired properties remains challenging. In this study, we introduce a flexible reinforcement learning method for flow-matching based generative models, allowing the velocity field to be refined according to a user-defined reward function. In contrast to a pure conditional generation setup, where the set of conditions must be decided a priori, this framework allows fine-tuning of any unconditional or conditional model, reflecting a more realistic scenario where the target properties to be optimized often vary and are typically case-specific. This also enables joint optimization of continuous and discrete features in flow-matching models for the first time. Through extensive experiments across diverse optimization scenarios, we demonstrate that models trained with this strategy (*agents*) consistently outperform baseline approaches (*priors*) when evaluated against the target design criteria.

## Abstract (web summary)

Three-dimensional molecular generative models can design novel molecules but guiding them to meet specific property requirements remains challenging. Here, the authors introduce a flexible reinforcement learning method for flow-matching generative models, enabling dynamic fine-tuning and joint optimization of features, outperforming baseline approaches in diverse optimization scenarios.

## Introduction

The rapidly growing availability of high-resolution 3D protein structures of therapeutic interest has substantially accelerated innovation in structure-based drug design (SBDD), enabling systematic discovery of small molecules with tailored binding affinity and specificity^1^. With the target binding site identified, ligands can be selected from established molecular databases or designed de novo. The first option, known as virtual screening, involves filtering extensive chemical libraries to prioritize candidates for hit identification and lead optimization^2^. A typical screening approach evaluates favorable interactions by docking each compound into the binding pocket, requiring progressively greater computational resource at scale^3^. The suboptimal efficiency, accompanied by high false-positive rates^4^, limits the application of virtual screening to large-scale ligand campaigns^5,6^. To address these limitations, Artificial Intelligence (AI)-driven de novo generation have emerged as a promising paradigm for drug design^7–10^. Various generative architectures based on one-dimensional (e.g., SMILES) or two-dimensional molecular representations have been developed to explore chemical space, including variational autoencoders (VAEs)^10^, generative adversarial networks (GANs)^7^, and recurrent neural networks (RNNs)^8,11^.

On the other hand, many studies attempt to directly generate small molecules with AI models in 3D space^12–17^. Using continuous 3D coordinates as output alleviates difficulties observed in previous methods, such as unnatural ordering in autoregressive models or violations of the triangle inequality in graph-based models^14^. Among 3D generative architectures, diffusion and flow-matching based models have gained the most popularity^16–21^. Integrated with SE(3) equivariance as a natural inductive bias, these approaches have been leveraged in various models for target-aware molecular generation^18–23^. The simultaneous representations of ligand conformations and local environments facilitate the designs of compounds that more faithfully satisfy geometric and chemical constraints of protein pockets. Despite this advantage, independent evaluations report that ligands generated with 3D neural networks frequently exhibit low physical plausibility of binding pose prior to re-docking^24,25^. Although progress has been made to improve structural soundness, other challenges await for practical application of molecule generative models. Beyond on-target complementarity, drug discovery demands simultaneous satisfaction of numerous criteria, for example: solubility, metabolic stability, bioavailability, while avoiding toxicity^26^. In many cases, there is no methodological understanding of mechanism, and no agreed way to relate this to generated coordinates. As a consequence, these properties are often estimated either via data-driven models or heuristic rules^27^. Such case-specific requirements make the reinforcement learning (RL) formulations (e.g., REINVENT^9^) particularly advantageous, as they provide flexible control to optimize user-specified features alongside on-target complementarity. To enable widespread application of 3D generative methods in drug discovery, it is critical to develop strategies that allow simultaneous and flexible control over multiple other properties.

In other domains with distinguished data modalities, such as text and images, researchers have successfully employed guidance strategies to enhance controllability in diffusion or flow-matching based generative processes. The methods mainly fall into two categories: classifier-based guidance^28,29^ and classifier-free guidance^30,31^. Classifier-based guidance leverages gradients from external property predictors to steer generation. In the field of molecular generation, there are similar implementations, such as a fragment-distance constraining term for linker design^32^, and a neural network parameterized predictor for target property^33,34^. Qiu et al.^35^ proposed a guidance method for Bayesian Flow Networks that refines the sampling process by evaluating an energy function on previous states. These methods rely on differentiable, well-performing functions to provide necessary gradients. However, obtaining such functions either by empirical formulation or additional training is nontrivial. Most optimization objectives in the pharmaceutical industry are not explicitly defined with respect to quantitative features and are thus non-differentiable (e.g., outcomes of physical based simulations or external predictive models). Moreover, constructing well-curated, labeled datasets for training predictors is costly. These factors limit the generalization of classifier-based guidance to broader optimization tasks in molecular design. In contrast, classifier-free guidance achieves comparable performance in image generation. It linearly combines the conditional and unconditional score estimates during fine-tuning, thereby bypassing the necessity of gradients. Hu et al.^36^ applied the method to improve a series of molecular properties using a pre-trained diffusion model, where optimizing each property required a corresponding conditional model. While effective for single-objective optimization, the approach can be challenging to extend to multiple properties optimization (MPO) tasks. Classifier-free guidance treats all conditions with the same weight, and is therefore unable to balance inherently competing objectives. Related to our work, several molecular generation guidance methods do not require differentiable score functions. Zhou et al.^37^ proposed an optimization algorithm based on a decomposable diffusion model for molecular generation, which iteratively improves generation quality by providing better prior fragments decomposed from high-scoring molecules generated in the previous round. AliDiff^38^ and DecompDPO^39^ construct the fine-tuning loss function based on user-defined preferences, which are derived from the pairwise rewards of winning and losing ligands.

Here, we present a reinforcement learning (RL) framework for flow-matching (FM) generative models. The methodology aims to fine-tune a FM generative model (the *agent*), based on a pre-trained FM generative model (the *prior*). By integrating reward functions as weighting factors throughout the training process, our agent model is able to produce larger proportion of compounds exhibiting specified properties. The rewards can arise from a wide variety of score functions without the necessity of being differentiable. This is also the first method to support fine-tuning of FM models on both continuous and discrete features. We systematically validate the performance of agent models across diverse molecular properties, including under MPO scenarios. Results demonstrate that this RL framework can effectively improve pre-trained models’ capabilities to generate samples with targeted properties. We summarize our contributions as follows:

- We propose a flexible reinforcement learning method for flow-matching based generative models, allowing the velocity field to be refined according to user-defined rewards, including non-differentiable objectives.
- To our knowledge this is the method first to jointly optimize continuous and discrete flows in FM models, a critical capability for generating molecules that have continuous coordinates and discrete chemical features (atom and bond types).
- A learnable molecular-size sampler is incorporated into the training process to determine the more appropriate number of atoms for a given context.
- Through extensive experiments in both unconditional and conditional settings, we demonstrate that RL-trained agent models consistently outperform prior models on target objectives.

## Results

We first provide a theoretical description of our RL-training method for FM based molecular generative models. We then evaluate our method across a range of application scenarios. The goal is to show the efficiency and versatility of this approach to transform a pre-trained flow-matching model into agent models tailored for specific tasks, such as improving one or several molecular properties. The assessments for these properties are either through established computation chemistry software, or additionally trained machine learning models (e.g. for property prediction). For all experiments, we first normalize each scalar property to the [0, 1] range and then aggregate them by computing their geometric mean (see Section 4 for further details). After training the agent, we generated samples from both the prior and the agent for comparison.

The pre-trained model we chose as PRIOR model is SemlaFlow^17^, a state-of-the-art FM model parametrized by a set of weights *θ*_*prior*_ and denoted by ${p}_{prior}^{\theta }$, which is capable of unconditionally sampling molecules in 3D space. The goal of RL fine-tuning is to improve the ability of the prior model to generate molecules that satisfy a set of predefined criteria, which are summarized by a single reward function *r*(*z*) ∈ [0, 1], where *z* denotes a molecule. Intuitively, we expect that the generated data distribution associated with the agent will be proportional to the reward, that is:

1 $${q}_{agent}(z)\propto {q}_{prior}(z)r(z).$$

To enforce this distribution shift, we modify the SemlaFlow loss by introducing the reward as a weighting factor, analogous to importance sampling^40^, thereby emphasizing high-reward regions during training. The fine-tuning is stopped as soon as the agent scores are no longer increasing. As is commonly observed in RL training, the agent model may suffer from policy collapse by overemphasizing high-reward regions^41,42^. To mitigate this issue, we add a regularization term that restricts the parameters of the agent model to remain close to those of the prior model. The number of atoms used to generate each batch of molecules is sampled from a learnable distribution that is updated based on rewards at each epoch. The adaptive mechanism offers the agent greater flexibility to select context-appropriate molecular sizes.

In the next section, we provide results of applying this method to controllable 3D molecular generation across four kinds of property-steered tasks:

- Intrinsic property optimization: The agent is rewarded by polar surface area computed by RDKit, to assess method performance on physicochemical properties;
- Customized property optimization: The agent is rewarded using predicted activities from a pre-trained model, to assess method performance on task-specific properties;
- Dual-objective optimization: The agent is rewarded by a score combining two objectives, to assess method’s generalization to MPO tasks;
- Conditional generation: The agent is rewarded by interaction energy based on ligand binding pose within a specified pocket, to assess method performance under conditional generation scenarios.

### Intrinsic property optimization

**Fig. 1:**
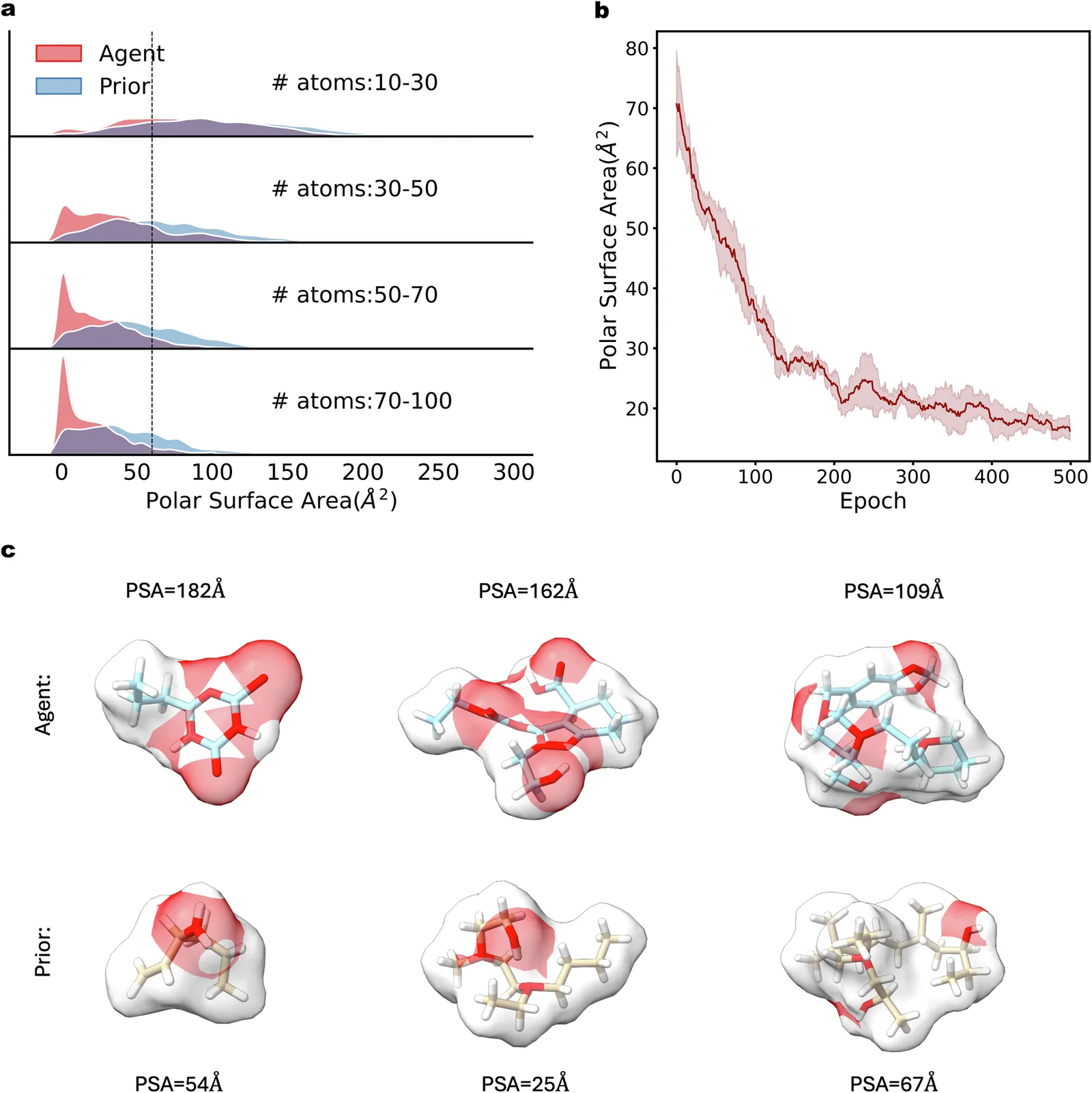
Results of fine-tuning with the objective of minimizing Polar Surface Area (PSA). **a** Distribution of PSA values across varying molecular sizes (number of atoms). Red and blue represent samples from the agent and prior models, respectively. After removing invalid structures, samples were grouped into four sets for visualization. **b** Temporal progress of model performance, illustrated by batch-median PSA value over training epochs. The solid line represents the average over 5 runs, and the shaded region indicates the standard deviation. **c** Representative pairs of molecules generated from identical initial noise with agent (top) and prior (bottom). Three columns from left to right correspond to number of atoms: 20, 40, 60. The general molecular surfaces are rendered in white, while the polar surface areas in red. Numeric labels adjacent to each structure indicate the corresponding PSA values. The best agent from the 5 runs is used to show the results in (**a**) and (**c**).

In this experiment, we aim to minimize the polar surface area (PSA) of the generated compounds. The PSA refers to the total solvent-accessible surface of polar atoms and associated hydrogens in a molecule. As one of the key physicochemical properties for drug development, PSA significantly influences absorption, permeability, and the ability of molecules to traverse blood-brain barriers^43^. Compounds with high PSA values often exhibit reduced absorption and limited membrane permeability, restricting their pharmacokinetic profiles. We trained the agent under RL with the objective of rewarding generated compounds with lower PSA and number of atoms ranging between 10 and 100. Upon convergence (epoch 300; see Fig. 1b), 5000 molecules were generated from both the prior and the agent. During inference, the number of atoms for the agent model is sampled from the atom number distribution learned during the RL training, while the number of atoms for the prior model is sampled uniformly between 10 and 100.

Table 1 compares the performance of the prior and agent models when optimizing PSA, in terms of PSA (in Å^2^) and validity (ratio of valid molecules generated by the model), uniqueness (ratio of unique molecules generated by the model), QED (Quantitative Estimate of Druglikeness), and *E*_*strain*_ (strain energy per atom in kcal/mol/atom). The agent achieves a substantially lower PSA (9.00 Å^2^ vs. 67.51 Å^2^), and higher validity (89.8% vs. 85.8%) than the prior, while maintaining the same QED (0.54). This improvement comes at a slight cost strain energy (*E*_*strain*_ of 3.85 kcal/mol/atom vs. 2.27 kcal/mol/atom), reflecting a trade-off between pose quality and optimized molecular properties. From a more qualitative angle, Figure 1a depicts that across all range of molecular sizes, the agent shows an enhanced ability to generate molecules with lower PSA. Structural analysis of pairs of molecules generated by two models further support this observation (see Figure 1c). Although both models yielded structurally similar compounds given the same molecular size, the agent consistently produced molecules with lower PSA.

**Table 1:** Property evaluations for molecules in the training set, generated by the prior model and the agent model

Model	Validity *↑*	Uniqueness *↑*	QED *↑*	E_strain_*↓*	PSA *↓*
Trainset(10–100)	100.0%	100.0%	0.49 ± 0.23	0.35 ± 0.26	119.12 ± 44.52
Prior	85.8%	98.1%	0.54 ± 0.21	2.27 ± 3.83	67.51 ± 47.30
Agent	89.8 ± 0.8%	98.7 ± 0.4%	0.54 ± 0.01	3.85 ± 0.6	39.00 ± 1.11

The agent is trained using PSA (in Å^2^) as reward function. Values for the agent model are reported as averages over 5 runs, with standard deviations. For each run, 5000 molecules are generated. Values for the prior model are given as average and standard deviation from a single sampling of 5000 molecules. Values for the training set are computed from 5000 molecules with atom number ranging from 10 to 100, randomly sampled from GEOM-Drugs. Validity represents the proportion of valid samples among all samples; uniqueness is calculated among valid samples; the other metrics are shown as averages over valid samples.

### Customized property optimization

The aim of this experiment is to steer the prior towards chemical regions which increase the biological activity of compounds binding to specific receptors. Such a property is influenced by multiple factors, from shape complementarity to polarity, thus hard to be determined by empirical rules. Therefore, we adopted a predictive neural network model used in ref. ^44^ to predict probability of given molecule being active to DRD2 receptor, using it both as a reward function during RL training and as an evaluation tool during validation. During training, we found that maximizing *p*(active∣molecule) (abbreviated as *p* in figures and tables for clarity) might induce a suboptimal strain energy performance for the agent model. Therefore, we included the minimization of *E*_*strain*_ as another component in the score function for training (see Supplementary Information Section 2).

**Fig. 2:**
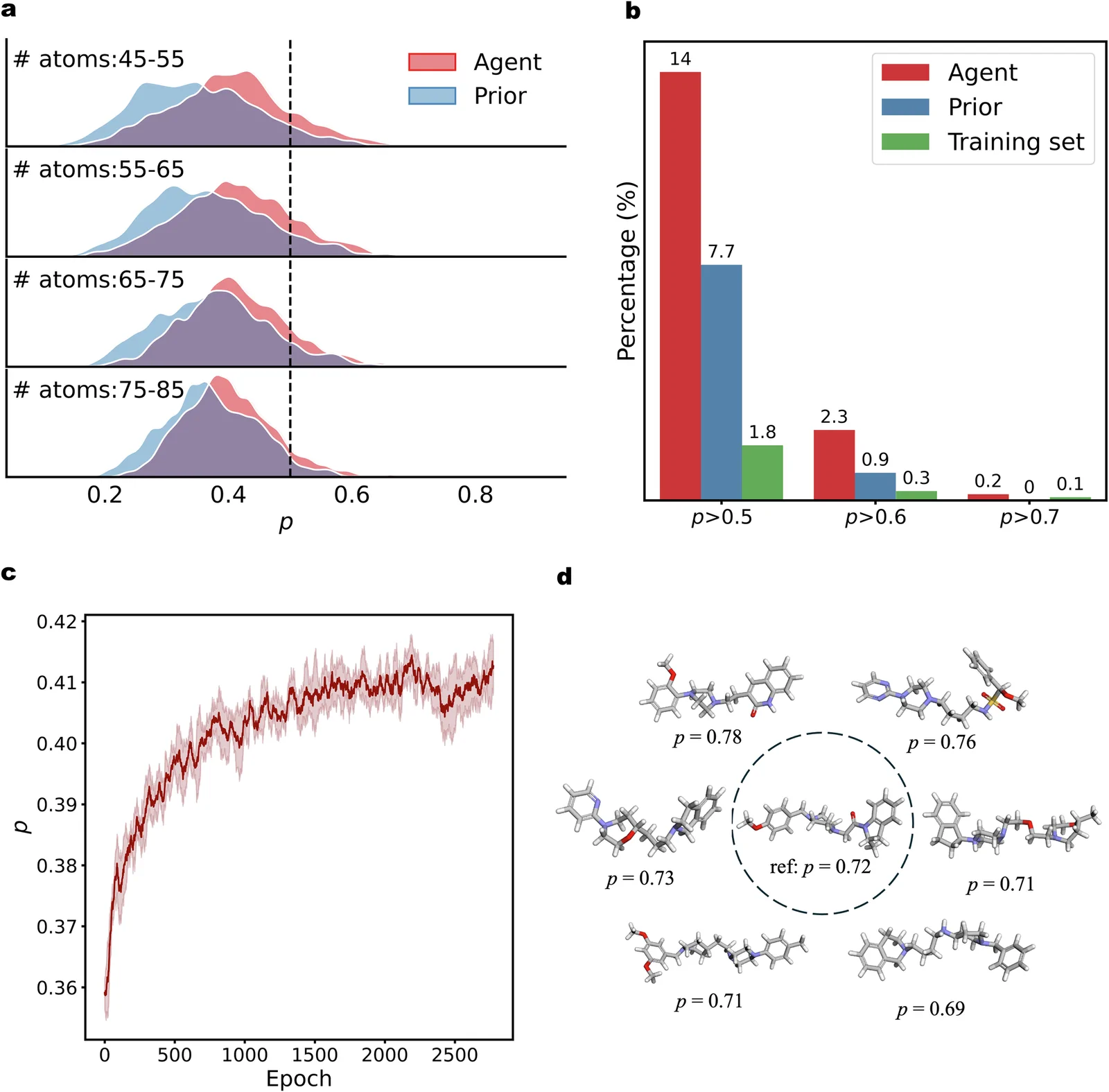
Results of fine-tuning with the objective of jointly maximizing *p* and minimizing *E*_*strain*_. **a** Distribution of predicted activity *p* across different molecular sizes (number of atoms). Red and blue represent samples from the agent and prior models, respectively. After removing invalid structures, samples were grouped into four sets for visualization. **b** Comparative proportions of molecules with elevated *p* values across three different dataset. Red, blue and green denote data sampled from the agent model, the prior model, and the GEOM-Drugs dataset used for prior model training, respectively. **c** Temporal progress of model performance, illustrated by batch-median *p* over training epochs. The solid line shows the average over 5 runs, and the shaded region indicates the standard deviation. **d** Representative top-scoring molecules generated by the agent model. The best agent from the 5 runs is used to show the results in (**a**), (**b**) and (**d**). All depicted molecules were energy-minimized using the MMFF94 force field for visualization purposes.

For evaluation, we generated 5000 molecules each from the prior and the agents (epoch 1000; see Fig. 2c), and analyzed the distributions of predicted bioactivity *p*(active∣molecule) along with other intrinsic molecular properties such as validity, uniqueness, QED and *E*_*strain*_. Table 2 reports the results. We observe that although the agent’s strain energy increases, validity increases from 84.1% to 90.8%, uniqueness remains at 100%, average QED increases from 0.54 to 0.59, and average predicted activity *p* rises from 0.37 to 0.41. Figure 2a shows the distributions of *p*(active∣molecule) for the prior and agent across four different ranges of molecular sizes, illustrating that the agent generates a higher proportion of predictive bioactive molecules.

**Table 2:** Property evaluations for molecules in the training set, generated by the prior model and the agent model

Model	Validity *↑*	Uniqueness *↑*	QED *↑*	E_strain_*↓*	*p↑*
Trainset (45–85)	100.0%	100.0%	0.47 ± 0.20	0.34 ± 0.18	0.30 ± 0.09
Prior	84.1%	100.0%	0.54 ± 0.19	1.85 ± 2.84	0.37 ± 0.09
Agent	90.8 ± 0.8%	100.0 ± 0.0%	0.59 ± 0.01	2.27 ± 0.47	0.41 ± 0.00

The agent model is trained by jointly maximizing predicted *p* and minimizing *E*_*strain*_. Values for the agent model are reported as averages over 5 runs, with standard deviations. For each run, 5000 molecules are generated. Values for the prior model are given as average and standard deviation from a single sampling of 5000 molecules. Values for the training set are computed from 5000 molecules with atom number ranging from 45 to 85, randomly sampled from GEOM-Drugs. Validity represents the proportion of valid samples among all samples; uniqueness is calculated among valid samples; other metrics are shown as averages over valid samples.

*p* refers to *p*(active∣molecule).

Figure 2b depicts the percentage of molecules generated by the agent and the prior, as well as in the original SemlaFlow training set, that have *p*(active∣molecule) greater than 0.5, 0.6, and 0.7. The agent generates almost two times active molecules than the prior, and over seven times more than the training set. We also visualized in Figure 2d six representative top-scoring molecular structures generated by the agent. They demonstrated similar shapes with an already known active molecule (dashed circled in the center), which was selected from predictive neural network’s training data ExCAPE-DB^45^ with pIC50 = 5.7, *p*(active∣molecule) = 0.72 and QED = 0.8^44^.

### Dual-objective optimization

Optimization of candidate compounds in drug development aims to satisfy multiple, often conflicting, properties that must be balanced to achieve good solutions^46^. In de novo design, such multi parameter optimization (MPO) is typically addressed either through stepwise selection after sampling or by integrating multiple objectives into a single loss function during training (e.g., classifier-free methods). In this section, we demonstrate the ability of our reinforcement learning framework to steer the agent to optimize a user-defined MPO problem.

To evaluate the effectiveness of our reinforcement learning strategy for MPO, we conducted a dual-property experiment as an empirical demonstration. Three agents were independently trained with RL loops using different reward functions (see Section 4.4 for details of score transforms): (1) targeting LOGP value near 2.0; (2) maximizing *p*(active∣molecule); (3) simultaneously optimizing both LOGP and *p*(active∣molecule) with the same goals in (1) and (2). For each of the agents, we generated 5000 molecules using converged model (epoch 1000) for quality comparison. Results are shown in Table 3. All three agents achieved better validity, same uniqueness and better QED compared to the prior model. Although agents trained with *p* as score function component show a moderate increasing in strain energy, it can be improved by including *E*_*strain*_ into score function as shown in Section 2.2.

**Table 3:** Property evaluations for molecules in training set, generated by the prior model and the agent model

Model	Validity *↑*	Uniqueness *↑*	QED *↑*	E_strain_*↓*	*p↑*	logP
Trainset (45–85)	100.0%	100.0%	0.47 ± 0.20	0.34 ± 0.18	0.30 ± 0.09	3.36 ± 2.15
Prior	84.1%	100.0%	0.54 ± 0.19	1.85 ± 2.84	0.37 ± 0.09	4.01 ± 1.75
Agent (only logP)	90.2 ± 0.9%	100.0 ± 0.0%	0.57 ± 0.01	2.23 ± 0.36	0.36 ± 0.01	2.44 ± 0.12
Agent (only *p*)	92.1 ± 0.4%	100.0 ± 0.0%	0.61 ± 0.00	3.87 ± 0.47	0.41 ± 0.00	3.70 ± 0.13
Agent (both *p* and logP)	92.5 ± 0.6%	100.0 ± 0.0%	0.62 ± 0.01	4.53 ± 0.74	0.41 ± 0.00	2.45 ± 0.14

*p* is defined in Section 2.2. Values for the agent models are reported as averages over 5 runs, with standard deviations. For each run, 5000 molecules are generated. Values for the prior model are given as average and standard deviation from a single sampling of 5000 molecules. Values for the training set are computed from 5000 molecules with atom number ranging from 45 to 85, randomly sampled from GEOM-Drugs. Validity represents the proportion of valid samples among all samples; uniqueness is calculated among valid samples; other metrics are shown as averages over valid samples.

*p* refers to *p*(active∣molecule).

**Fig. 3:**
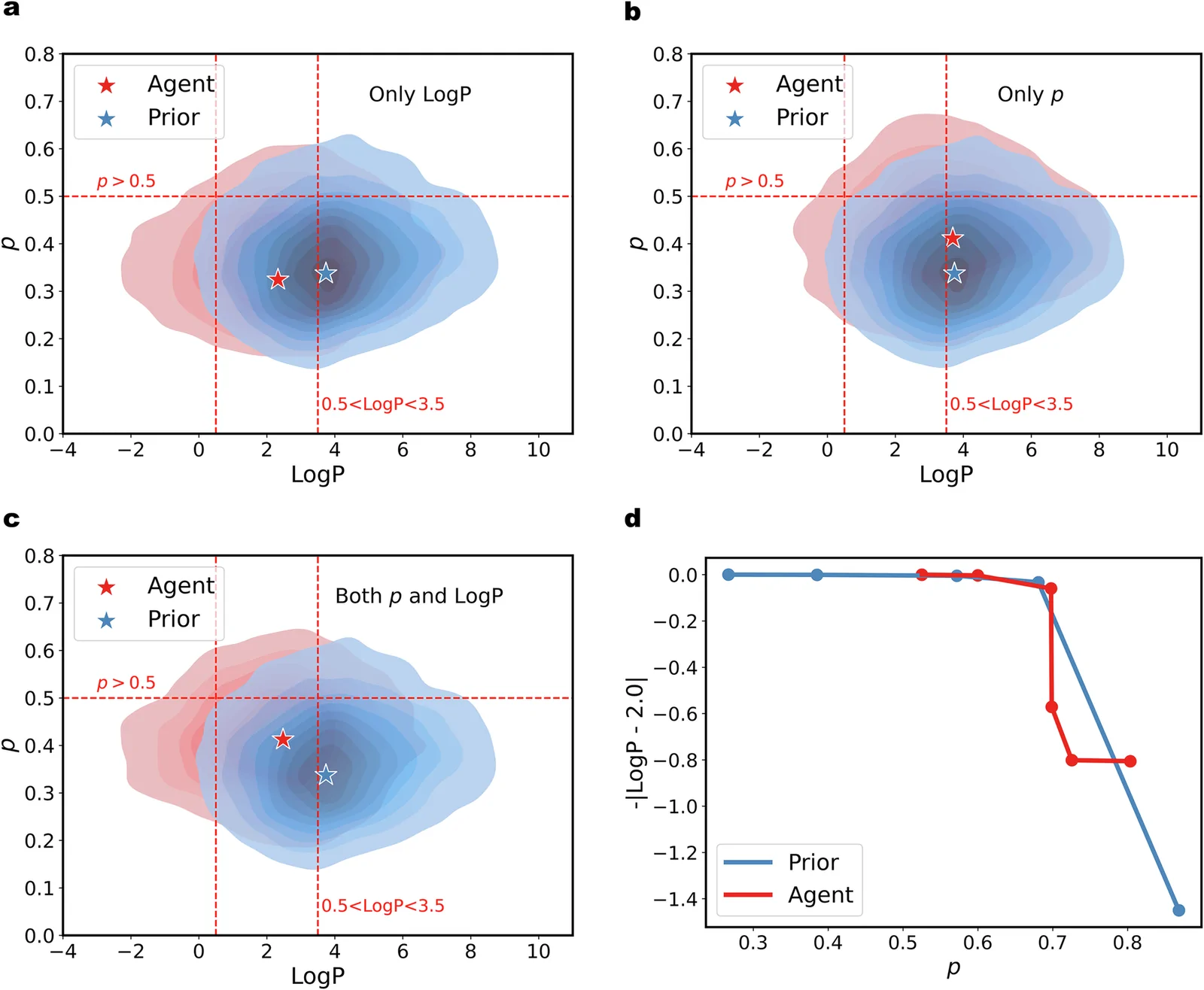
Results of optimizing both LOGP and *p*(active∣molecule) (abbreviated as *p* in figure for clarity). Two-dimensional distribution of *p* and LOGP values for molecules generated by the agent and prior models, where the agent was trained using **a** only LOGP, **b** only *p*, **c** both LOGP and *p* as the objective function. In all three panels, density centers of the agent and prior generated samples are indicated by red and blue dots, respectively. The best agent from the 5 runs is used to show the results. Only valid molecules were used in the plot. **d** Pareto frontiers corresponding to the two datasets depicted in panel (**c**). The x-axis denotes *p*, while the y-axis represents the negative absolute deviation of a sample’s LOGP value from +2.0, which approximates the optimal LOGP for drug-like compounds.

Regarding the target objectives, our comprehensive assessment shows that both LOGP and *p*(active∣molecule) improve after RL training, though to different extents across agents. The agents trained on a single reward component achieve the highest performance for their target property, with averaged *p* of 0.41 (case 2) and averaged LOGP of 2.44 (case 1), respectively (see Table 3). Their performance on the other property either decreases moderately (see Fig. 3a) or remains largely unchanged (see Fig. 3b). In contrast, the agent fine-tuned with a dual-objective function (case 3) improves both properties (see Fig. 3c). There are 346 out of 5000 molecules generated by the agent in case 3 with *p* > 0.5 and LOGP between 0.5 and 3.5, which almost doubles the amount generated by the prior model (176 out of 5000). RL-training notably expands the diversity and coverage of generated molecules that satisfy desirability criteria with respect to both endpoints (see Fig. 3d).

### Conditional generation

Although our approach has shown the ability to optimize agents for different properties, its application has so far been limited to unconditional ligand generation. To assess its efficacy in protein-conditioned tasks, we fine-tuned a conditional variant of SemlaFlow^47^. The original unconditional SemlaFlow was modified to incorporate pocket coordinates as conditions at the stage of training and inference (see Method 4).

Here, the RL training objective is to generate ligands adopting favorable binding poses, which from a potential energy perspective corresponds to producing bound ligands with more negative intermolecular interaction energies—specifically, electrostatic and van der Waals contributions—denoted as $\Delta {{{{\mathcal{E}}}}}_{{{{\rm{atom}}}}}$. Since energy standards vary between receptors, we chose a specific receptor human uridine-cytidine kinase 2 (PDBID: 6N53) and fine-tuned the agent to minimize $\Delta {{{{\mathcal{E}}}}}_{{{{\rm{atom}}}}}$ within its pocket. Energy scores were computed using the MMFF94 molecular mechanics force field implemented in PyTorch (https://github.com/MolecularAI/TorchMMFF94), and the molecular size range was set to match the number of atoms of the native bound ligand. We note that while this energetic term covers some types of protein-ligand interaction, it does not consider energy differences between bound and unbound states, and therefore is not a binding energy. Moreover, since entropic contributions are not considered, it does not represent a free energy difference either. However, it serves as a simple model of protein-pose complementarity for demonstration purposes and it is lightweight to evaluate. During the experiments, we found that the agent learned to reduce $\Delta {{{{\mathcal{E}}}}}_{{{{\rm{atom}}}}}$ by sampling poses with higher strain energy *E*_*strain*_. Therefore, we trained our conditional agent using both $\Delta {{{{\mathcal{E}}}}}_{{{{\rm{atom}}}}}$ and *E*_*strain*_ as the dual-objective reward function.

**Fig. 4:**
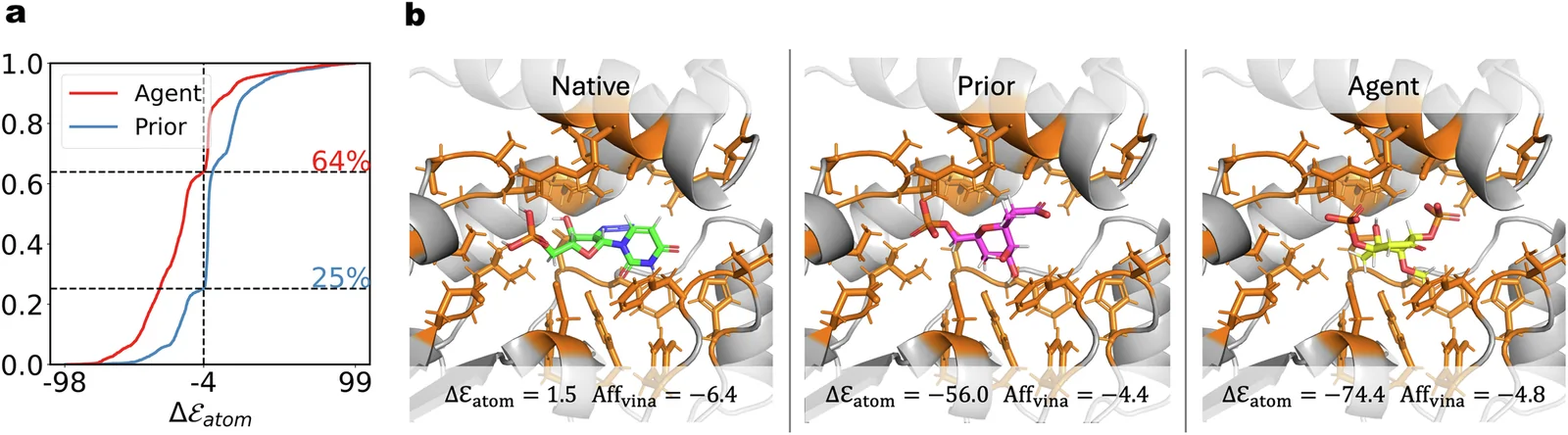
Results of optimizing interaction energy in a conditional generation task based on the binding pocket of uridin-cytidine kinase 2 (PDBID: 6N53). **a** Cumulative distribution plot of interaction energy $\Delta {{{{\mathcal{E}}}}}_{{{{\rm{atom}}}}}$ for prior and agent models. **b** Visualization of three molecules: native ligand in PDB, ligand generated by the prior, and ligand generated by the agent. Interaction energies ($\Delta {{{{\mathcal{E}}}}}_{{{{\rm{atom}}}}}$) and binding affinities estimated by AutoDock Vina (Aff_vina_) are labeled.

The overall quality of the generated molecules from the agent remains close to the prior, as shown in Table 4. Figure 4a reports the cumulative distribution of $\Delta {{{{\mathcal{E}}}}}_{{{{\rm{atom}}}}}$ for the prior and the agent. Molecules with positive or zero interaction energies indicate potential steric clashes or suboptimal interactions with the pocket. After RL training, the proportion of ligand with negative interaction energies increased substantially (see Fig. 4a). Individual cases comparing ligand poses generated by different models provide further insights into the performance of the agent (see Fig. 4b). The molecules generated by the agent can better adapt to the local pocket in terms of less steric clashes, and more aligned shape orientations resulting in better predicted affinities by docking.

**Table 4:** Property evaluations for molecules in training set, generated by the prior model and the agent model

Model	Validity *↑*	Uniqueness *↑*	QED *↑*	E_strain_*↓*	$\Delta {{{{\mathcal{E}}}}}_{{{{\rm{atom}}}}}$*↓*
Trainset (25–45)	100.0%	100.0%	0.70 ± 0.16	0.37 ± 0.32	38.76 ± 25.39
Prior	95.3%	99.6%	0.49 ± 0.17	4.63 ± 4.14	2.00 ± 22.48
Agent	87.7 ± 0.8%	98.7 ± 0.2%	0.42 ± 0.01	4.01 ± 0.31	−15.80 ± 0.59

The agent model is trained using both $\Delta {{{\mathcal{E}}}}\,{\mbox{atom}}\,$ and *E*_*strain*_ as the reward function. Values for the agent model are reported as averages over 5 runs, with standard deviations. For each run, 5000 molecules are generated. Values for the prior model are given as average and standard deviation from a single sampling of 5000 molecules. Values for the training set are computed from 5000 molecules with atom number ranging from 25 to 45, randomly sampled from GEOM-Drugs. Validity represents the proportion of valid samples among all samples; uniqueness is calculated among valid samples; other metrics are shown as averages over all valid samples; $\Delta {{{{\mathcal{E}}}}}_{{{{\rm{atom}}}}}$ is shown as the average over valid samples with value smaller than 100 kcal/mol/atom.

## Discussion

3D generative models have recently emerged as promising tools to design drug-like candidates, as they are capable to directly generate binders within the spatial context of protein pocket. Nevertheless, the practical utility of these models has often been limited by issues such as unrealistic binding poses and suboptimal chemical properties in the generated molecules. In this work, we propose an efficient reinforcement learning framework for 3D molecular generation. Leveraging a fast-sampling flow-matching model, we developed a reinforcement learning framework which steers agents to produce molecules with specified properties based on predictive models, physiochemical properties and physics-informed scores. The agent training process integrates online rewards of real-time evaluators and regularization of the prior model, allowing systematic optimization of both intrinsic and customized molecular properties. Across all tested cases, our fine-tuned agents consistently outperformed the priors, enlarging the proportion of molecules that satisfies targeted design criteria.

Although approaches like classifier guidance^33,34^ or classifier-free guidance have obtained notable success in domains such as image and text generation, their application to chemical design remains limited. Due to the sparsity of labeled data and the vast designable chemical space, training reliable, differentiable predictors as property classifiers is not feasible in all cases. Additionally, directly using the target property as a training condition introduces challenges for MPO tasks^36^. Our method combines a reward-weighted FM model with RL process, alleviating the need to train an extra classifier and facilitating the swift transformation from a baseline model to specialized agent models tailored for diverse objectives.

Recently, policy-based RL techniques have been utilized in directed molecule generation^48^. Theoretically, our approach is equivalent to them in terms of the goal of maximizing the expected rewards. However, unlike the necessity of computing trajectory likelihoods, which are either time-consuming or inaccurate for discrete representations, our method exerts direct control over the velocity field during RL training, bypassing the hybrid likelihood estimation of generated molecular samples.

Prior advances in fine-tuning flow-based generative models have focused on differentiable reward functions^49^. However, in molecular design, many critical features are assessed using knowledge-driven criteria. For such cases, deriving gradients is infeasible. Our approach addresses this gap by relying solely on relative outcomes from property evaluators, which is crucial for applying de novo generative models to real-world applications. Future work includes reducing the possible degeneration in non-target properties by imposing better regularization, and incorporating stochasticity into sampling to enhance the agent’s exploration ability.

## Methods

### Flow-matching models

Flow-matching (FM) models^50,51^ are continuous-time generative models that learn to sample new data points *x*_1_ ~ *p*_1_ (e.g. images), by transporting samples from a simple known distribution *x*_0_ ~ *p*_0_ (e.g. standard Gaussian) along a time-dependent probability path *p*_*t*_. Note that *p*_1_ is unknown and we only have access to samples. This process is described by a continuous time-dependent flow *ψ*_*t*_ that maps an initial point *x*_0_ at *t* = 0 into intermediate points in times, i.e. *x*_*t*_ = *ψ*_*t*_(*x*_0_) ~ *p*_*t*_, and for *t* = 1 we have *x*_1_ = *ψ*_1_(*x*_0_). The flow is defined by a time-dependent velocity field *u*_*t*_ and satisfies the Ordinary Differential Equation (ODE):

2 $$\frac{d}{dt}{\psi }_{t}(x)={u}_{t}({\psi }_{t}(x)),$$

where *u*_*t*_ generates the probability path *p*_*t*_ allowing to flow from a simple distribution *p*_0_ to the data distribution *p*_1_. The data distribution *p*_1_ can be reconstructed by integrating the velocity *u*_*t*_ over *t* ∈ [0, 1].

The target velocity field *u*_*t*_(*x*_*t*_) and the intermediate distributions *p*_*t*_ are generally intractable^50^. To address this, we instead consider a conditional velocity field *u*_*t*_(*x*_*t*_∣*x*_1_) which depends on a data point *x*_1_. *p*_*t*_ is then constructed by marginalizing Gaussian conditional probability paths ${p}_{t| 1}(x| {x}_{1})={{{\mathcal{N}}}}(x| {\mu }_{t}({x}_{1}),{\sigma }_{t}{({x}_{1})}^{2}I)$, where *x*_1_ denotes an individual data point sampled from *p*_1_. For the corresponding flow written as *ψ*_*t*∣1_(*x*) = *σ*_*t*_(*x*_1_)*x* + *μ*_*t*_(*x*_1_), the conditional vector field can be derived in a closed form, that is:

3 $${u}_{t}({x}_{t}| {x}_{1})=\frac{{\sigma }_{t}^{{\prime} }({x}_{1})}{{\sigma }_{t}({x}_{1})}(x-{\mu }_{t}({x}_{1}))+{\mu }_{t}^{{\prime} }({x}_{1}).$$

A possible choice for mean and standard deviation that change linearly over time is^50^:

4 $${\mu }_{t}({x}_{1})=t\,{x}_{1}\,,\quad {\sigma }_{t}({x}_{1})=1-t.$$

In this case, the closed form conditional velocity simplifies to ${u}_{t}({x}_{t}| {x}_{1})=\frac{{x}_{1}-{x}_{t}}{1-t}$. The FM ${u}_{t}^{\theta }({x}_{t})$ (parametrized by *θ*) models the conditional velocity, and it is trained to minimize the following loss

5 $${{{{\mathcal{L}}}}}_{{{{\rm{CFM}}}}}(\theta )= {{\mathbb{E}}}_{t,{x}_{t},{x}_{1}}\parallel {u}_{t}^{\theta }({x}_{t})-{u}_{t}({x}_{t}| {x}_{1}){\parallel }^{2}, \\ \,{{\mbox{where}}}\,t \sim U[0,1],{x}_{t} \sim {p}_{t| 1}(\cdot | {x}_{1}),{x}_{1} \sim {p}_{1}.$$

FM is further extended to be capable of generating categorical data^52,53^, referred to as Discrete Flow Matching (DFM). It builds a probability flow by using Continuous Time Markov Chains (CTMCs) to describe jump activities among states. Combination of continuous and discrete flow facilitate the multimodal generative model for molecules.

#### FM for 3D molecular design

In this work, we consider SemlaFlow^17^ a state-of-the-art FM model for de novo generation of 3D molecules. Each molecule is represented as a tuple *z*_1_ = (*x*_1_, *a*_1_, *b*_1_, *c*_1_) of atomic coordinates ${x}_{1}\in {{\mathbb{R}}}^{3}$, atomic types ${a}_{1}\in {{{\mathcal{A}}}}$, bond types ${b}_{1}\in {{{\mathcal{B}}}}$, and formal charges ${c}_{1}\in {{{\mathcal{C}}}}$, respectively. ${{{\mathcal{A}}}}$, ${{{\mathcal{B}}}}$, and ${{{\mathcal{C}}}}$ are discrete sets defining the possible atom, bond, and charge types. Instead of predicting velocity field *u*_*t*_, the SemlaFlow model *f*_*θ*_ is trained to directly predict data points ${\hat{z}}_{1}=({\hat{x}}_{1},{\hat{a}}_{1},{\hat{b}}_{1},{\hat{c}}_{1})={f}_{\theta }({z}_{t})$ given *z*_*t*_. Note that ${\hat{a}}_{1}$, ${\hat{b}}_{1}$, and ${\hat{c}}_{1}$ are probability vectors. At inference time, the noise is first sampled from the scaled distribution ${p}_{noise}^{\pi }({z}_{0}| m)$ where *m* > 0 represents the number of atoms. For the coordinates, an Euler solver is used to integrate the ODE following:

6 $${x}_{t+\Delta t}={x}_{t}+\frac{{\hat{x}}_{1}-{x}_{t}}{1-t}\Delta t,$$

where *Δt* is the step size. The categorical variables are sequentially sampled using the strategy described by Campbell et al.^52^ which requires the current state (e.g. *a*_*t*_) and the predicted state (e.g. ${\hat{a}}_{1}$). The final loss function for training SemlaFlow can be written as:

7 $${{{\mathcal{L}}}}(\theta )={\lambda }_{x}\parallel {\hat{x}}_{1}-{x}_{1}{\parallel }^{2}+{\lambda }_{a}{a}_{1}\log ({\hat{a}}_{1})+{\lambda }_{b}{b}_{1}\log ({\hat{b}}_{1})+{\lambda }_{c}{c}_{1}\log ({\hat{c}}_{1})$$

8 $${{{{\mathcal{L}}}}}_{{{{\rm{SemlaFlow}}}}}(\theta )={{\mathbb{E}}}_{t,{z}_{t},{z}_{1}}\left[{{{\mathcal{L}}}}(\theta )\right],$$

where *t* ~ *U*[0, 1], *z*_*t*_ ~ *p*_*t*∣1_( ⋅ ∣*z*_1_), *z*_1_ ~ *p*_1_. The loss function uses weighting factors of *λ*_*x*_ = 1.0, *λ*_*a*_ = 0.2, *λ*_*b*_ = 1.0, and *λ*_*c*_ = 1.0, matching the default values used in the original SemlaFlow work^17^.

### Reinforcement learning framework

Given a trained SemlaFlow model, denoted as the prior *f*_*θ*_, our goal is to fine-tune a copy of this model, ${f}_{{\theta }{{{\rm{agent}}}}}$ (referred to as the agent), to generate molecules that satisfy a set of desired properties. These properties are represented by a reward function *r*(*z*_1_) ∈ [0, 1], where values near 0 correspond to low scores and values near 1 to high scores. Within this reinforcement learning framework, the generative distribution is adjusted so that molecules with higher scores are assigned higher probabilities. Formally, given a reward function *r*(*z*_1_) our goal is to re-weight the probability distribution of the agent as

9 $${p}_{{{{\rm{agent}}}}}({z}_{1})=\frac{r({z}_{1})p({z}_{1})}{\int\,r({z}_{1})p({z}_{1})d{z}_{1}}$$

for which we adapt the loss strategy of refs. ^54,55^, generalizing it (and proving it in Supplementary Information Section 1) to categorical variables, that is

10 $${{{{\mathcal{L}}}}}_{{{{\rm{RL}}}}}({\theta }_{{{{\rm{agent}}}}})={{\mathbb{E}}}_{t,{z}_{t},{z}_{1}^{{{{\rm{agent}}}}}}\left[r({z}_{1}^{{{{\rm{agent}}}}}){{{\mathcal{L}}}}({\theta }_{{{{\rm{agent}}}}})\right],$$

where ${z}_{1}^{{{{\rm{agent}}}}}$ is generated by the agent, ${f}_{{\theta }_{{{{\rm{agent}}}}}}$ and ${{{\mathcal{L}}}}({\theta }_{{{{\rm{agent}}}}})$ is the loss defined in Eq. (7) but applied to the agent’s weights.

### Adaptive molecular size

The molecular generation process with SemlaFlow requires to specify the molecular size, i.e. the number of atoms *m*. The molecular size directly influences both novelty and quality of the generated ligands. To remove the bias introduced by manual selection, the molecular size is sampled from an adaptive probability distributions which is jointly learned with *θ*_agent_. Each size *m* is associated with an independent Beta distribution parameterized by (*α*, *β*). At the beginning of each epoch, we sample once from each of the *m* Beta distributions and *m* to the index corresponding to the largest sample (i.e., the argmax). After generation, the Beta distribution corresponding to the selected size *m* is updated based on the average reward ${\bar{r}}^{(i)}$ obtained in epoch *i*. Specifically, after each epoch, the parameters are updated as follows

11 $${\alpha }^{(i+1)}={\alpha }^{(i)}+{\bar{r}}^{(i)}\quad {\beta }^{(i+1)}={\beta }^{(i)}+1-{\bar{r}}^{(i)}.$$

This formulation increases the expected value of the Beta distribution for molecular sizes that consistently achieve higher rewards while preserving stochasticity in the selection process.

### General reward function settings

Different molecular properties exhibit heterogeneously distributed scores; for example, the QED score lies in [0, 1], while docking scores span ${\mathbb{R}}$. To ensure comparability, raw scores are normalized to (0, 1) using a sigmoid, a reverse sigmoid, or a double sigmoid transformation. The sigmoid is used when higher scores above a threshold are desirable, the reverse sigmoid is applied when lower scores are preferable, and the double sigmoid is employed to reward scores falling within a specific range. The sigmoid function is defined as

12 $$\sigma (s(z);{k}_{1},{b}_{1})=\frac{1}{1+{e}^{-{k}_{1}(s(z)-{b}_{1})}},$$

where *s*(*z*) represents a raw score, *k*_1_ and *b*_1_ denote the slope and center of the sigmoid function, respectively. The reverse *σ*_*r*_ and double sigmoid *σ*_*d*_ are defined as

$${\!\!}{\sigma }_{r}(s(z);{k}_{2},{b}_{2}){\!}={\!}1-\sigma (s(z);{k}_{2},{b}_{2}),{\,\,} {\sigma }_{d}(s(z);{k}_{1},{k}_{2},{b}_{1},{b}_{2}){\!}={\!}\sigma (s(z);{k}_{1},{b}_{1})-\sigma (s(z);{k}_{2},{b}_{2}).$$

These parameters (*k*_1_, *k*_2_, *b*_1_, and *b*_2_) directly control the steepness and shift of the mapping, thereby modulating sensitivity of the reward function. Their values are selected empirically, based on the prior distribution of molecular properties and desired training speed. For *n* scores components *s*_1_, …, *s*_*n*_, the total reward is defined as the weighted geometric averages of the all normalized scores

13 $$r(z)={\left[{\prod}_{i}{\sigma }_{i}{({s}_{i}(z))}^{{\omega }_{i}}\right]}^{1/{\sum }_{i}{\omega }_{i}}$$

where *σ*_*i*_ denotes a type among sigmoid, reverse sigmoid, or double sigmoid, *ω*_*i*_ > 0 denotes the weighting factor of corresponding component.

We compute the average reward by subtracting the batch-averaged reward $\bar{r}(z)$ from each individual reward *r*(*z*) within the batch. The resulting centered rewards serve as weighting factors during RL training, effectively emphasizing relative performance within the batch rather than absolute values. Empirically, we observed that subtracting the mean improves convergence.

### Regularization

To mitigate the issue of policy collapse in online training, we introduced a regularization term to constrain the agent model’s parameters proximal to those of the prior model:

14 $$\Omega ({\theta }_{{{{\rm{agent}}}}})={\lambda }_{r}\parallel {\theta }_{{{{\rm{agent}}}}}-\theta {\parallel }^{2},$$

where *θ*_agent_ and *θ* denote agent and prior parameters, respectively. *λ*_*r*_ is a tunable weighting coefficient, which we fixed *λ*_*r*_ = 0.1 in all our experiments. This term is added to the RL training loss function to penalize large deviations from the prior model.

### Experimental details

#### Prior models

For all the unconditional RL training tasks reported in this work, we employed the same pre-trained SemlaFlow model as the prior. This model was trained on QM9^56,57^ and GEOM^58,59^ datasets and contains ~40 million learnable parameters. Sampling from the prior was performed under default hyperparameters and implementation described by Irwin et al.^17^.

#### Agent models

Agent model parameters were initialized from the prior. All agents are trained with Adam optimizer with a learning rate of 1e−5 and gradient-norm clipping at 1.0. Agents generate a single batch for each RL training epoch, with size of 64 in unconditional experiments and size of 16 in conditional experiments.

Within each training epoch, the agent first samples a batch of molecular candidates in a forward pass without gradient tracking. The final outputs are then converted into molecular objects, which are subsequently used for score computation. Intermediate states *z*_*t*_ are obtained via interpolation between initial noise state and terminal state. ${z}_{1}^{\theta }$ is predicted from the randomly sampled timestep *t* and its corresponding interpolated state *z*_*t*_. The agent model’s parameters are updated by minimizing a weighted loss between ${z}_{1}^{\theta }$ and sampled *z*_1_, weighted by the rewards calculated from the scores *r*(*z*_1_). The training process is summarized in Section 4.6.4.

For each score function, we run the RL training experiments for five times with different random seeds.

#### Score functions

In this section we provide a full description of the score functions we have used during RL training.

*p*(active∣molecule) estimates the probability that a generated molecule is active (pIC50 ≥ 5) against the dopamine receptor type 2 (DRD2) receptor. It is predicted by a pretrained model from ref. ^44^, which is based on a random-forest architecture and trained on data from ExCAPE-DB^45^. The predictor is implemented in the same way as in ref. ^60^. To be noted, since the predictive model accepts molecular graphs defined by atomic types and their connectivity, all generated 3D structures were first converted into SMILES strings before evaluation.

Polar Surface Area (PSA) quantifies the contribution of polar atoms to the solvent accessible surface area (SASA) of a molecule. SASA is computed using atomic van der Waals radii from the periodic table. The subset of SASA associated with polar atoms (Nitrogen, Oxygen, Sulfur and Phosphorus) is extracted with RDKit (v2026.3.5) package rdFreeSASA, and the sum of these contributions is reported as PSA value.

Log*P* represents the logarithm of the partition coefficient between octanol and water, widely used as a measurement of molecule’s lipophilicity. It’s predicted by the Crippen model^61^ implemented within RDKit (v2026.3.5).

$\Delta {{{{\mathcal{E}}}}}_{atom}$ measures the per-atom interaction energy for a ligand with *m* atoms in pose *z* relative to a protein pocket ${{{\mathcal{P}}}}$:

15 $$\frac{{{{{\mathcal{E}}}}}_{{{{\rm{bound}}}}}(z| {{{\mathcal{P}}}})-{{{{\mathcal{E}}}}}_{{{{\rm{unbound}}}}}(z)}{m},$$

where ${{{{\mathcal{E}}}}}_{{{{\rm{bound}}}}}(z| {{{\mathcal{P}}}})$ is the ligand’s potential energy evaluated in the presence of the protein pocket, including only ligand intramolecular terms plus ligand-protein nonbonded interactions (van der Vaals and electrostatics), and ${{{{\mathcal{E}}}}}_{{{{\rm{unbound}}}}}(z)$ is the ligand’s intramolecular energy in the same pose without protein. Energies are computed using the MMFF94^62^ force field implemented with PyTorch. Pocket is extracted by selecting residues that have at least one atom within 3.5 Å of the native bound ligand.

Aff_vina_ refers to the standard AutoDock Vina^63^ scores. It’s computed with the raw generated poses and the native receptor protein in GNINA (v1.3.1)^64^ without re-docking. The protein is preprocessed by Schrödinger (v2025.3)’s PrepWizard for geometric correction and appropriate protonation.

Validity is the proportion of generated molecules that correspond to chemically valid single fragments, and that are successfully sanitized within RDKit (v2026.3.5).

Uniqueness measures the fraction of generated molecules that are unique in their SMILES representation within the valid generated dataset.

QED denotes the quantitative estimate of drug-likeness score implemented in RDKit (v2026.3.5).

E_strain_ measures the potential energy difference of the same molecule before and after geometry optimization with MMFF94 force field. Reported value is normalized by the number of atoms. It provides a global assessment about the structural quality of the generated 3D conformations.

#### Training process

In Algorithm 1, we summarized the RL training process of agent models given a prior SemlaFlow model.

##### Algorithm 1

**Training process**

**Input:***α*_*m*_ = 1.0, *β*_*m*_ = 1.0, *N*, *λ*_*r*_ = 0.1, prior SemlaFlow model *f*_*θ*_, *η* learning rate

1: $${f}_{{\theta }_{{{{\rm{agent}}}}}}$$ = *f*_*θ*_

2: **for** *n* ∈ {1, …, *N*} **do**

3:  $$m={{{{\rm{arg}}}}\, {\max}}_{m}({p}_{m})$$, where *p*_*m*_ ~ Beta(*α*_*m*_, *β*_*m*_)

4:  Sample a batch of molecules ${z}_{1} \sim {f}_{{\theta }_{{{{\rm{agent}}}}}}$

5:  Sample time steps *t* ~ *U*(0, 1)

6:  Interpolate *z*_*t*_ given *t*, *z*_1_, and *z*_0_ ~ *p*_0_

7:  $${z}_{1}^{{{\rm{agent}}}}={f}_{{\theta }{{{\rm{agent}}}}}({z}_{t})$$

8:  Compute the regularized RL objective (Eq. (10)):

$${{{{\mathcal{L}}}}}_{{{{\rm{RL}}}}}({\theta }_{{{{\rm{agent}}}}})={{\mathbb{E}}}_{t,{z}_{t},{z}_{1}^{{{{\rm{agent}}}}}}\left[r({z}_{1}){{{\mathcal{L}}}}({\theta }_{{{{\rm{agent}}}}})\right]+{\lambda }_{r}\parallel {\theta }_{{{{\rm{agent}}}}}-\theta {\parallel }^{2}$$

9:  Update agent model’s parameters:

$${\theta }_{{{{\rm{agent}}}}}={\theta }_{{{{\rm{agent}}}}}-\eta {\nabla }_{\theta }{{{{\mathcal{L}}}}}_{{{{\rm{RL}}}}}({\theta }_{{{{\rm{agent}}}}})$$

10:  Update Beta distributions (Eq. (11)):

$${\alpha }_{m}={\alpha }_{m}+\bar{r},\quad {\beta }_{m}={\beta }_{m}+1-\bar{r}$$

11: **end for**

12: **return** Fine-tuned agent model ${f}_{{\theta }_{{{{\rm{agent}}}}}}$

## Supplementary information

Supplementary Information

Transparent Peer Review file

Description of additional supplementary word file

Supplementary Data 1

ML- Reporting-Summary

## Data Availability

Source data of this paper are provided in Supplementary Data.

## Code availability

Code of this paper is available at https://github.com/MolecularAI/flow_matching_rl^65^. FlowMol3 source code and pretrained model checkpoint are publicly available at https://github.com/Dunni3/FlowMol. The implementation of the DRD2 activity prediction model is available at https://github.com/r1cc4r2o/weiss, and the corresponding model checkpoint can be obtained from https://github.com/MolecularAI/transformer_rl.
