# Supplementary material for: Controllable molecular generation with fine-tuned flow-matching model
Source: Commun Chem. 2026 Sep 5;9:291. doi: 10.1038/s42004-026-02188-z (PMC13546285; doi:10.1038/s42004-026-02188-z)
Supplement: Supplementary file 1 [file 42004_2026_2188_MOESM1_ESM.pdf]

Supplementary Information: Controllable molecular generation with fine-tuned flow-matching model

Kunyu Wang[1]*, Jon Paul Janet[1], Alessandro Tibo[1]
[1]Molecular AI, Discovery Sciences, R&D, AstraZeneca, Gothenburg, Sweden

Contents

1 Generalized Reinforcement Learning loss strategy **2**

2 Results of optimizing both predicted activity and strain energy **3**

3 Comparison to a baseline method **3**

4 Performance on other type of prior model **3**

5 Regularization weighting coefficient λ_r **5**

6 Comparison against other loss functions **6**

*Corresponding author: kunyu.wang@astrazeneca.com

1 Generalized Reinforcement Learning loss strategy

In this section, we proposed a generalized loss function for fine-tuning Flow-matching models involving both continuous and discrete variables. Each of our data sample is represented as a tuple $z = (x, h)$ of continuous variable $x \in \mathbb{R}^3$ and categorical variable $h \in \mathcal{H}$, respectively. $\mathcal{H}$ is a discrete set of S categories. We use matrices $p^{\theta}_{1|t}$ of size $D \times S$ to represent the categorical probabilities predicted by the neural network at $t = 1$ given h_t, where D is the number of constituted atoms.

Assuming x and h are independently distributed, i.e. $p(z) = p(x) \cdot p(h)$, loss function (defined in Equation 8 in the main text) of optimizing θ can be obtained by combining the continuous velocity loss in Equation 5 and the categorical loss defined by Campbell et al. (2024):

$$\mathcal{L}_{\text{SemlaFlow}}(\theta) = \mathbb{E}_{t\sim U[0,1], z_t\sim p_{t|1}(\cdot|z_1), z_1\sim p(z_1)}\big[\mathcal{L}(\theta)\big] \tag{1}$$

Given a reward function $r(z_1)$, our goal is to re-weight the probability distribution of the agent such as

$$p_{\text{agent}}(z_1) = \frac{r(z_1)p(z_1)}{\int r(z_1)p(z_1)dz_1} \tag{2}$$

From now on we use Z to denote the constant denominator, i.e. $Z = \int r(z_1)p(z_1)dz_1$. By adding $r(z_1)$ as weight into Equation 1 we obtain:

$$\begin{aligned}
\mathcal{L}_{RL}(\theta_{\text{agent}}) &= \mathbb{E}_{t\sim U[0,1], z_t\sim p_{t|1}(\cdot|z_1), z_1\sim p(z_1)}\big[r(z_1)\mathcal{L}(\theta_{\text{agent}})\big] && (3)\\
&= \int_0^1 \int_{z_1} \int_{z_t} r(z_1)\mathcal{L}(\theta_{\text{agent}})p(z_1)p_{t|1}(\cdot|z_1)\, dt\, dz_1\, dz_t && (4)\\
&= \int_0^1 \int_{z_1} \int_{z_t} r(z_1)p(z_1)\mathcal{L}(\theta_{\text{agent}})p_{t|1}(\cdot|z_1)\, dt\, dz_1\, dz_t && (5)\\
&= \int_0^1 \int_{z_1} \int_{z_t} Z \cdot p_{\text{agent}}(z_1)\mathcal{L}(\theta_{\text{agent}})p_{t|1}(\cdot|z_1)\, dt\, dz_1\, dz_t && (6)\\
&= Z \int_0^1 \int_{z_1} \int_{z_t} p_{\text{agent}}(z_1)\mathcal{L}(\theta_{\text{agent}})p_{t|1}(\cdot|z_1)\, dt\, dz_1\, dz_t && (7)\\
&= Z \cdot \mathbb{E}_{t\sim U[0,1], z_t\sim p_{t|1}(\cdot|z_1), z_1\sim p_{\text{agent}}(z_1)}\big[\mathcal{L}(\theta_{\text{agent}})\big]. && (8)
\end{aligned}$$

Since Z is constant, it does not affect the optimization process. Therefore, minimizing Equation 3 is equivalent to minimizing Equation 1 with $z_1 \sim p_{\text{agent}}(z_1)$:

$$\nabla_{\theta_{\text{agent}}}\mathcal{L}_{RL}(\theta_{\text{agent}}) = Z \cdot \mathbb{E}_{t\sim U[0,1], z_t\sim p_{t|1}(\cdot|z_1), z_1\sim p_{\text{agent}}(z_1)}\big[\nabla_{\theta_{\text{agent}}}\mathcal{L}(\theta_{\text{agent}})\big]. \tag{9}$$

2 Results of optimizing both predicted activity and strain energy

Supplementary Table 1: Molecular generation results comparing different agent models. Agent model is trained using only predicted activity p as reward function (first row), or using both predicted activity p and E_{strain} as reward function (second row). Validity represents the proportion of valid samples among all samples; uniqueness is calculated among valid samples; other metrics are shown as averages over valid samples.

Model	Validity ↑	Uniqueness ↑	QED ↑	E_{strain} ↓	p ↑
Only p	$92.1 \pm 0.4\%$	$100.0 \pm 0.0\%$	0.61 ± 0.00	3.87 ± 0.47	0.41 ± 0.00
p and E_{starin}	$90.8 \pm 0.8\%$	$100.0 \pm 0.0\%$	0.59 ± 0.01	2.27 ± 0.47	0.41 ± 0.00

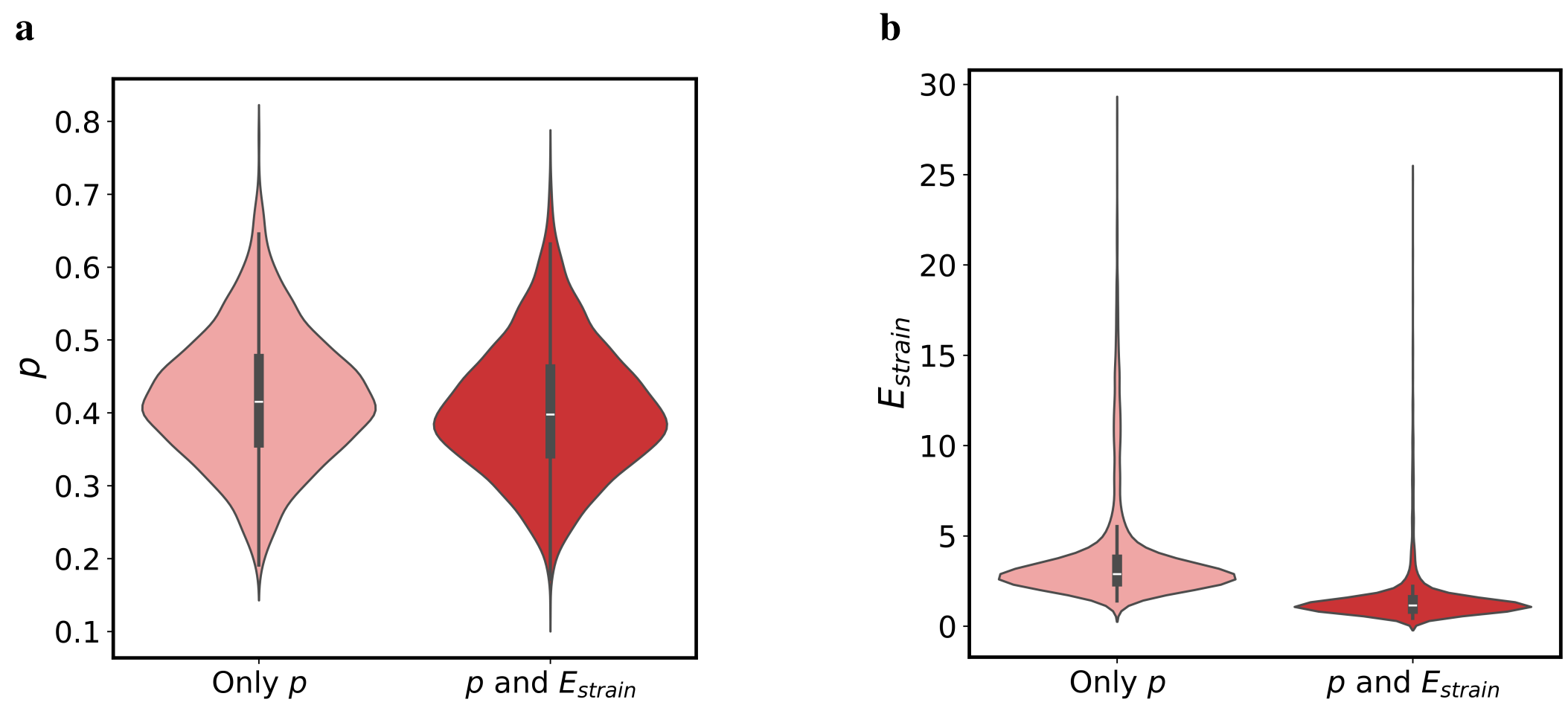

Supplementary Figure 1: Distributions of measured predicted bioactivity p (**a**) and strain energy E_{strain} (**b**) for valid molecules from 5,000 samples generated by agent trained with only p as objective (light red), or by agent trained with both p and E_{strain} as objective (dark red).

3 Comparison to a baseline method

We trained a baseline model using a two-stage fine-tuning strategy, as a comparison to our RL approach. In the first stage, the pre-trained model is used to sample 64,000 molecules. This choice mimics our RL strategy which generates 64 molecules for 1,000 steps. The top 10% scored ligands are stored as a dataset. In the second stage, this dataset is used to fine-tune the model for another 1,000 epochs with batch size 64. Inferences of the two models follow the same settings as in Section 2.2. The baseline model performs better in averaged $p(\text{active}|\text{molecule})$ and averaged strain energy per atom (Supplementary Table 2). However, the assessment of this two variables' collective distribution has shown that RL effectively reduces the proportion of extremely-high strain energy molecules, and promotes the upper bound of $p(\text{active}|\text{molecule})$ for generated molecules (Supplementary Figure 2).

4 Performance on other type of prior model

To demonstrate the generalizablity of our RL approach's application to other base generative models, we have conducted the RL training to another state-of-the-art model FlowMol Dunn & Koes (2026) and evaluated its performance. We chose the score function in Section 2.2 that involves $p(\text{active}|\text{molecule})$ and strain energy. RL-training has largely improved one of the target properties $p(\text{active}|\text{molecule})$ in terms of both average value and overall distributions across all atom numbers

Supplementary Table 2: Molecular generation results comparing different models. Agent model is trained using predicted activity p as reward function, where p is the abbreviation for $p(\text{active}|\text{molecule})$, the probability that a given molecule is active. For each model, 5,000 molecules are sampled from single run. Validity represents the proportion of valid samples among all samples; uniqueness is calculated among valid samples; other metrics are shown as averages over valid samples.

MODEL	VALIDITY ↑	UNIQUENESS ↑	QED ↑	E_{strain} ↓	p ↑
PRIOR	84.1%	100.0%	0.54 ± 0.19	1.85 ± 2.84	0.37 ± 0.09
AGENT (RL)	$90.8 \pm 0.8\%$	$100.0 \pm 0.0\%$	0.59 ± 0.01	2.27 ± 0.47	0.41 ± 0.00
FT-MODEL (BASELINE)	$88.3 \pm 0.9\%$	$100.0 \pm 0.0\%$	0.55 ± 0.01	1.95 ± 0.11	0.44 ± 0.00

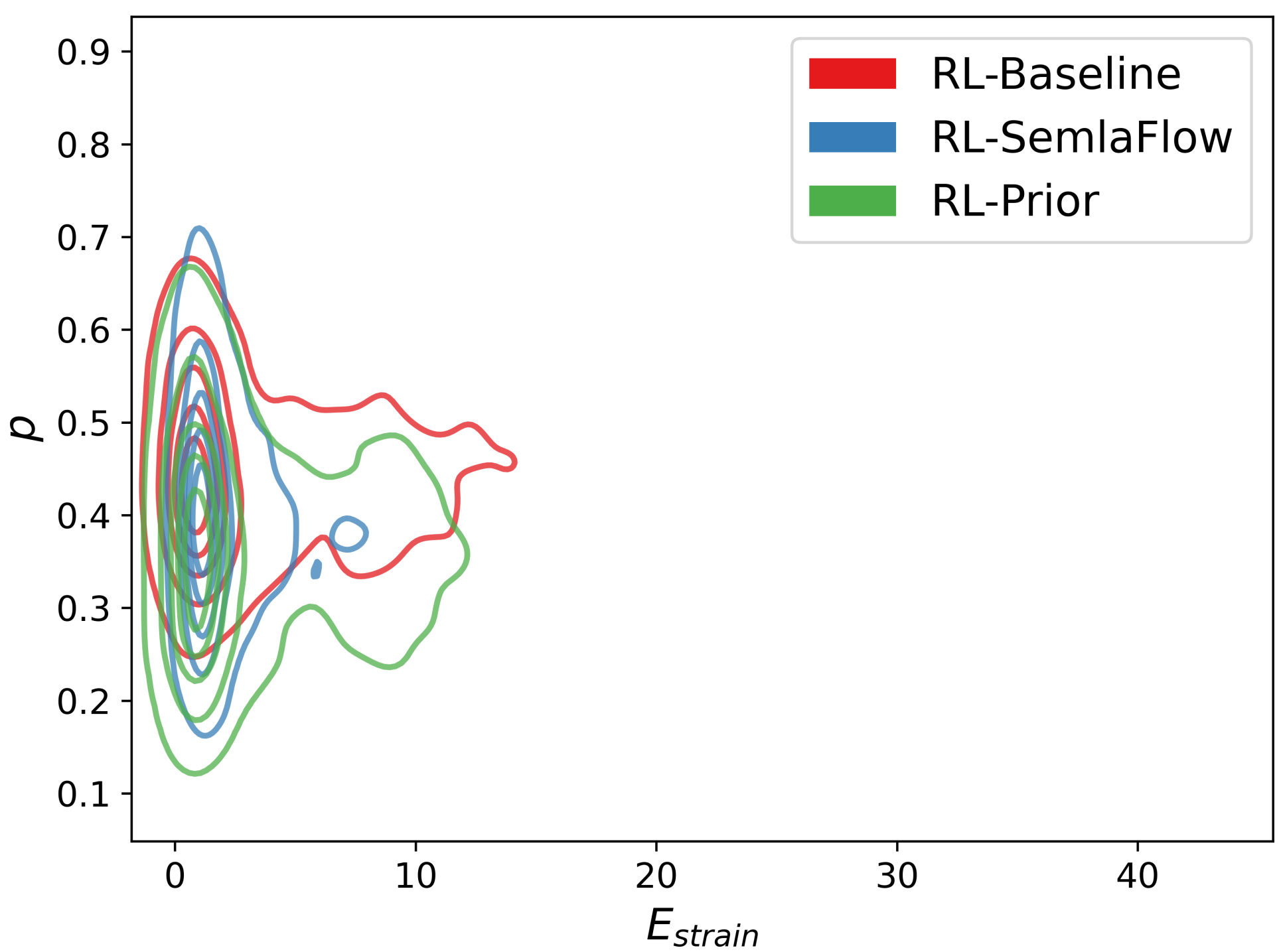

Supplementary Figure 2: 2D distributions of predicted bioactivity p and strain energy E_{strain} for valid molecules generated by three models.

Supplementary Table 3: Molecular generation results comparing different models. Agent model is trained using predicted activity p as reward function, where p is the abbreviation for $p(\text{active}|\text{molecule})$, the probability that a given molecule is active. For each model, 5,000 molecules are sampled from single run. Validity represents the proportion of valid samples among all samples; uniqueness is calculated among valid samples; other metrics are shown as averages over valid samples.

MODEL	VALIDITY ↑	UNIQUENESS ↑	QED ↑	E_{strain} ↓	p ↑
PRIOR-FLOWMOL	96.0%	100.0%	0.58 ± 0.18	0.52 ± 0.38	0.30 ± 0.09
RL-FLOWMOL	$94.7 \pm 3.1\%$	$100.0 \pm 0.0\%$	0.72 ± 0.02	0.92 ± 0.19	0.38 ± 0.01

(Supplementary Table 3, Supplementary Figure 3a). The other target property strain energy has been remained within acceptable range (Supplementary Table 3). After RL training, the agent model is able to produce 4 times of active ligands compared to the prior model (Supplementary Figure 3b).

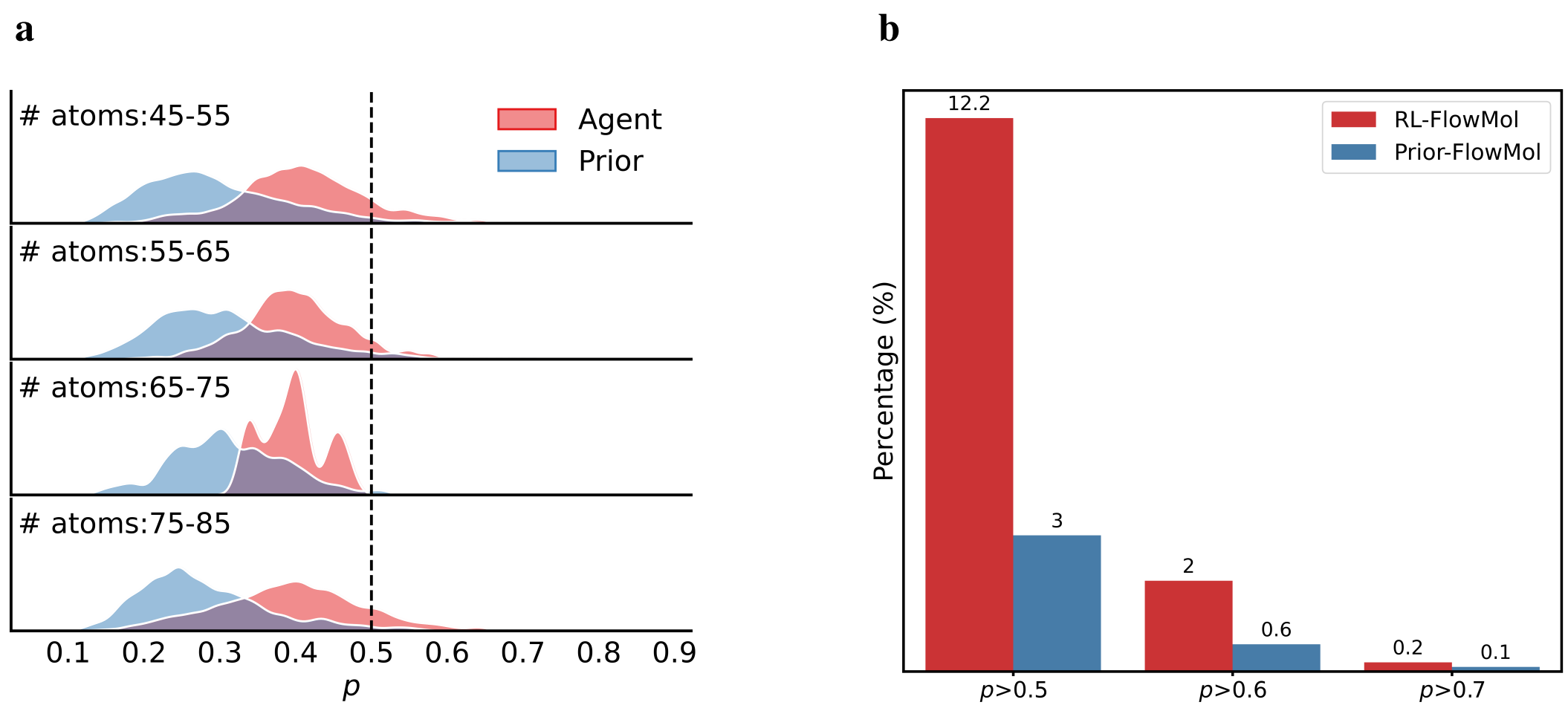

Supplementary Figure 3: Fine-tuning results of FlowMol model using score function in Section 2.2. **a.** Distribution of predicted activity p across different molecular sizes (number of atoms). **b.** Proportions of molecules in different p thresholds.

Note that the averaged QED of agent generated molecules has been increased for over 0.1, proving our approach's ability in preserving high quality ligands.

5 Regularization weighting coefficient λ_r

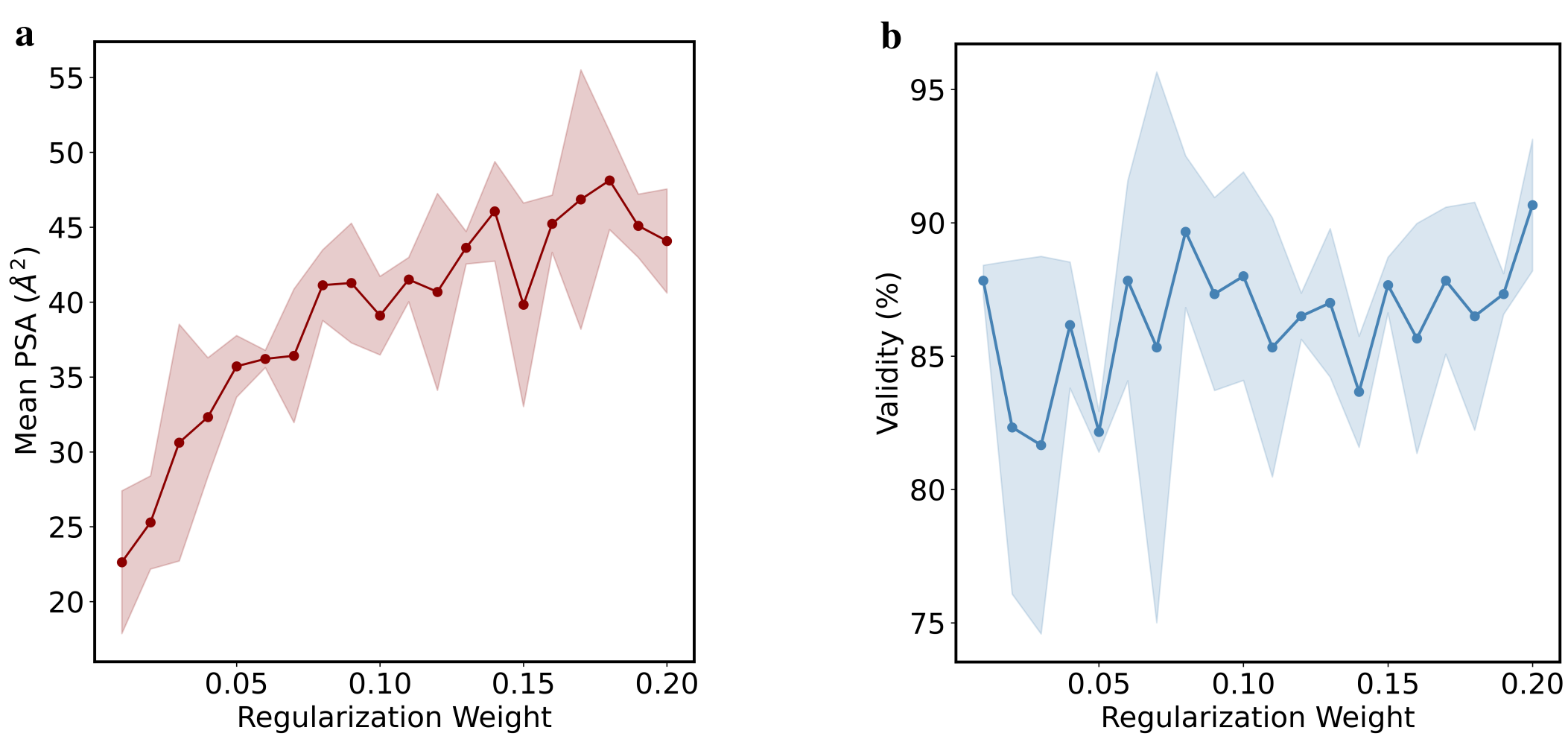

Supplementary Figure 4: Effect of various regularization weighting coefficient λ_r. Evaluation of (**a**) validity and (**b**) PSA for valid molecules generated by agents trained with different regularization coefficients λ_r from 0.01 to 0.2. Each dot represent the mean value averaged from 3 independent runs. Shades show the standard deviation.

To decide the appropriate value of weight coefficient λ_r of the regularization term, we trained a series of agents with various λ_r using PSA as the RL optimization objective. Experiment for each λ_r value was repeated for three times with different random seeds to give robust evaluations. With each of these agent models, 200 molecules were generated and compared for their mean PSA value and validity ratio (Supplementary Figure 4). Lower λ_r encourages the agent to perform better in target property. However, as λ_r approaching lower than 0.1, the validity tends to fluctuate, indicating a drop in model stability.

6 Comparison against other loss functions

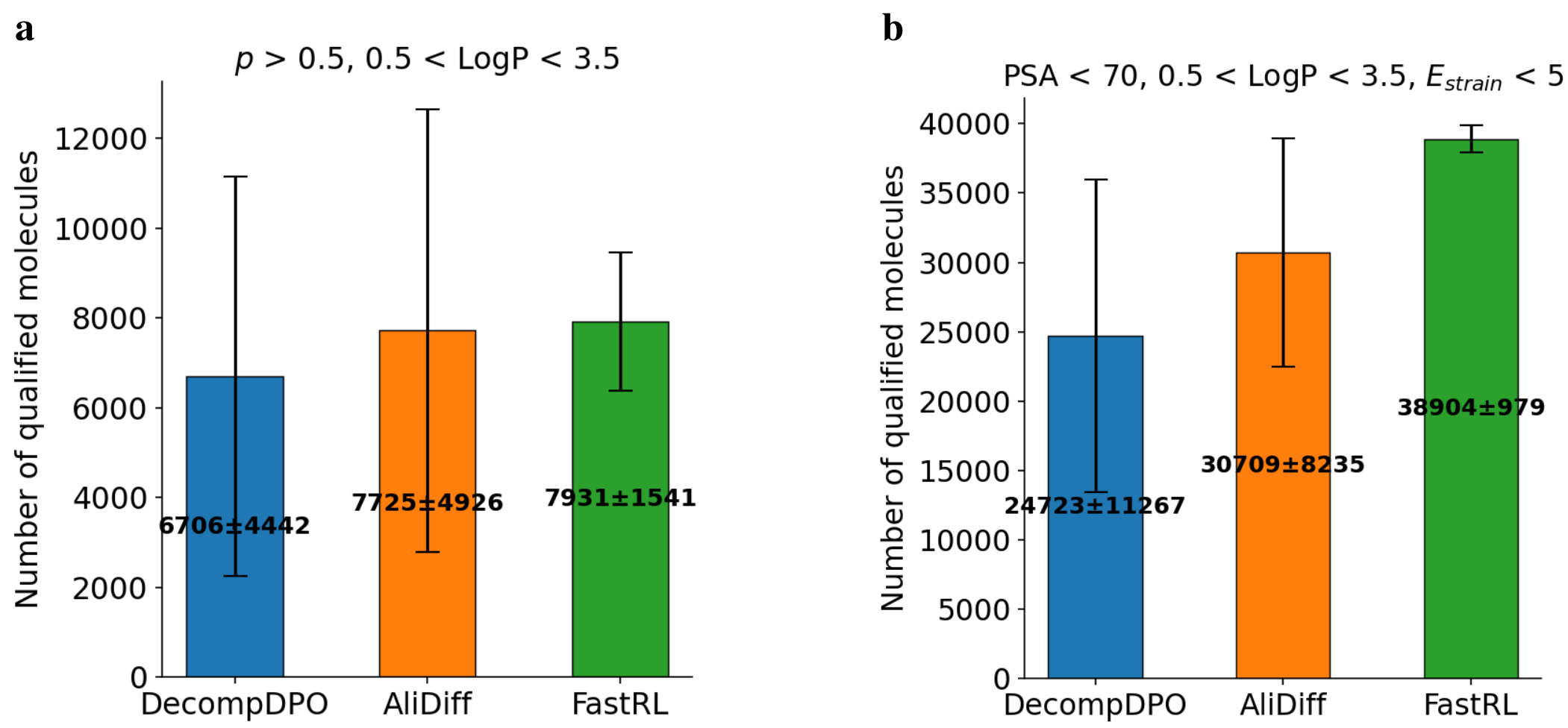

Supplementary Figure 5: Numbers of qualified molecules accumulated throughout 2,000 training epochs for three methods. Desiderata molecular properties are shown on top of each of plot. Bars and error bars represent the mean and standard deviation over five independent runs, respectively. **a** Agents were trained with the optimization goal of "Higher p, LOGP between 0.5 and 3.5" as in Section 2.3; **b** Agents were trained with the optimization goal of "Lower PSA, lower E_{strain}, and LOGP between 0.5 and 3.5".

To compare the performance of our RL method with other optimization approaches, we evaluated two additional algorithms, AliDiff Gu et al. (2024) and DecompDPO Cheng et al. (2026), under two optimization objectives and compared them with our method (FastRL). We applied their respective loss functions to fine-tune the same pretrained model used in our method. For each loss function, the agent was trained in an online RL setting following Algorithm 1, and the molecules sampled at each epoch were collected and evaluated. Supplementary Figure 5 shows the cumulative number of valid and qualified molecules generated by each method up to epoch 2,000. All three methods are able to generate molecules that meet the optimization requirements. On average, our method produced the largest number of qualified molecules with substantially lower variance, demonstrating its robustness across different optimization goals.

References

Andrew Campbell, Jason Yim, Regina Barzilay, Tom Rainforth, and Tommi Jaakkola. Generative Flows on Discrete State-Spaces: Enabling Multimodal Flows with Applications to Protein Co-Design, June 2024.

Xiwei Cheng, Xiangxin Zhou, Yuwei Yang, Yu Bao, and Quanquan Gu. Decomposed Direct Preference Optimization for Structure-Based Drug Design, February 2026.

Ian Dunn and David R. Koes. FlowMol3: Flow matching for 3D de novo small-molecule generation. *Digital Discovery*, 2026. doi: 10.1039/D5DD00363F.

Siyi Gu, Minkai Xu, Alexander Powers, Weili Nie, Tomas Geffner, Karsten Kreis, Jure Leskovec, Arash Vahdat, and Stefano Ermon. Aligning Target-Aware Molecule Diffusion Models with Exact Energy Optimization, October 2024.
