# Supplementary material for: Controllable molecular generation with fine-tuned flow-matching model
Source: Commun Chem. 2026 Sep 5;9:291. doi: 10.1038/s42004-026-02188-z (PMC13546285; doi:10.1038/s42004-026-02188-z)
Supplement: Supplementary file 2 [file 42004_2026_2188_MOESM2_ESM.pdf]

nature portfolio

Peer Review File

# Controllable Molecular Generation with Fine-tuned Flow-Matching Model

Corresponding Author: Dr Kunyu Wang

**This file contains all reviewer reports in order by version, followed by all author rebuttals in order by version.**

Version 0:

Reviewer comments:

Reviewer #1

(Remarks to the Author)
This paper studies an important problem of goal-directed molecular optimization. The authors fine-tune pre-trained flow mathcing model with RL to optimize molecular properties including intrinsic properties, customized properties, and multiple objectives.

Main comments.

1. The novelty is somewhat limited. The idea of fine-tuning diffusion models or flow matching have been explored in molecular generation [1]. The strategy for fine-tuning is simply an adaption from [2]

2. In section 2.3, how do you define the preference of different objectives.

3. The experiments are weak. The performance is only conducted between the prior model and the fine-tune model. It is important to compare the fine-tuned model with others state-of-the-art methods and apply the fine-tuning stategy to different prior models.

[1] Aligning Target-Aware Molecule Diffusion Models with Exact Energy Optimization

[2] Online Reward-Weighted Fine-Tuning of Flow Matching with Wasserstein Regularization

Reviewer #2

(Remarks to the Author)
This manuscript introduces a general framework for steering flow-matching generative models toward specific regions of chemical space. The proposed method allows a pre-trained model to be fine-tuned through a reinforcement learning procedure so that the generative distribution shifts toward molecules satisfying desired properties. Overall, the manuscript is clearly written, the problem statement is well motivated, and the methodology is easy to follow.

Strengths

- The approach is theoretically well motivated. The authors formulate the method in a principled framework by introducing reward-weighted fine-tuning of the flow-matching objective, allowing the velocity field of the generative process to be adjusted according to user-defined rewards.

The study considers not only single-property optimization but also multi-objective scenarios, including potentially conflicting objectives. Demonstrating the ability to steer generation under multiple constraints is an important aspect for practical molecular design tasks.

- The manuscript is clearly structured and easy to understand. The presentation of the method and experimental setup is accessible, making the work approachable for readers beyond specialists in generative modeling.

Points that should be addressed

- The experimental evaluation is somewhat limited in scope. The comparisons are primarily between the original prior model and the proposed reinforcement-learning-modified variant of the same model. This makes it difficult to assess the overall impact of the proposed steering technique relative to other modern generative models. Several recently developed 3D molecular generative models report stronger baseline performance than the architecture used here(eg. Megalodon Flow, FlowMol3, etc). Including comparisons with additional models, or at least discussing their expected behavior under the proposed framework, would strengthen the study.

- Even simple engineered baseline for steering could provide useful context. For example, one could consider resampling the training data based on the reward functon(or target property) and retraining the model. Such baselines would help isolate how much of the observed improvement arises specifically from the proposed reinforcement learning approach.

- Additional, ablation studies would also be helpful. Particularly, regarding the regularization term used to constrain the agent model toward the prior. Since this term is introduced to mitigate policy collapse and appears central to training stability, evaluating its influence on the final performance would clarify how sensitive the method is to this design choice. It can be justifyible to use it as it is if it is absolute standard of such models. In this case I would suggest change wording on it.

- Steering the model appears to introduce trade-offs in structural quality. In several experiments the validity decreases and strain energy increases compared to the prior model (in some error is increasing around 80% when validity errors increases from 12% to 20%). Also from the the results it is obvious that this degradation depend on the property itself (some exhibits larger drops). While this behavior is briefly mentioned, the manuscript would benefit from a more detailed discussion of these effects. In particular, it would be helpful to understand whether the degradation can be controlled by adjusting the regularization coefficient, modifying the reward function, or introducing additional structural constraints during training.

- The tables report only single average values without any indication of statistical uncertainty. Since the evaluation relies on stochastic sampling of generated molecules, reporting standard deviations or confidence intervals across multiple runs (or subsets) would make the comparisons more informative and scientific. Including reference values from the underlying dataset would also help contextualize some metrics. For example, reporting QED statistics for molecules within the desired property range in the training data would provide a useful baseline for interpreting the generated results.

- Some evaluation metrics would benefit from clearer explanation. In particular, the strain energy is reported in kcal/mol per atom. For typical drug-like molecules with tens of atoms, the reported values correspond to very large total strain energies (3 per atom means 150 for 50 atoms molecule, which is like bond breaking energy, while typical conformational energies do not exceed 5 kcal/mol). Clarifying how this metric is calculated and normalized would improve interpretability. It would also be helpful to ensure that the energy model used for evaluation is consistent with the one associated with the dataset or training setup, as inconsistent energy definitions can lead to misleading comparisons (https://doi.org/10.1039/D5DD00206K).

Overall assessment

Overall, this is a clearly written manuscript that presents a theoretically sound method for controllable molecular generation using flow-matching models. The proposed reinforcement learning framework is conceptually interesting and appears flexible enough to handle different types of objectives. With additional comparisons, ablation analyses, and clearer discussion of evaluation metrics and trade-offs, the work would be significantly strengthened. After addressing these points, the manuscript would likely be suitable for publication.

Version 1:

Reviewer comments:

Reviewer #1

(Remarks to the Author)
I appreciate the authors' responses, and I am satisfied with most of the clarifications provided. However, I still have a remaining concern regarding my last comment.

My point is that the proposed method should be compared with a broader range of property optimization methods. Specifically, to better demonstrate the advantage of the proposed algorithm over related optimization strategies, I suggest using the same pretrained prior model and applying different fine-tuning or optimization methods under a consistent setting. This would allow a more direct and fair comparison of the optimization capability of the proposed approach. Potential baselines to consider include AliDiff[1], DecompDPO[2], DECOMPOPT[3], and MolJO[4].

[1] Aligning target-aware molecule diffusion models with exact energy optimization
[2] Decomposed direct preference optimization for structure-based drug design
[3] CONTROLLABLE AND DECOMPOSED DIFFUSION MODELS FOR STRUCTURE-BASED MOLECULAR OPTIMIZATION

[4] Empower Structure-Based Molecule Optimization with Gradient Guidance

Reviewer #2

(Remarks to the Author)
I appreciate the authors' efforts in conducting additional experiments and ablation studies to address the concerns raised in my initial review. These concerns have been satisfactorily addressed, and I believe the manuscript is now ready for publication.

**Open Access** This Peer Review File is licensed under a Creative Commons Attribution 4.0 International License, which permits use, sharing, adaptation, distribution and reproduction in any medium or format, as long as you give appropriate credit to the original author(s) and the source, provide a link to the Creative Commons license, and indicate if changes were made.
In cases where reviewers are anonymous, credit should be given to 'Anonymous Referee' and the source.
The images or other third party material in this Peer Review File are included in the article's Creative Commons license, unless indicated otherwise in a credit line to the material. If material is not included in the article's Creative Commons license and your intended use is not permitted by statutory regulation or exceeds the permitted use, you will need to obtain permission directly from the copyright holder.
To view a copy of this license, visit https://creativecommons.org/licenses/by/4.0/

# Response to Reviewers: Controllable Molecular Generation with Fine-tuned Flow-Matching Model

Kunyu Wang, Jon Paul Janet, Alessandro Tibo

June 4, 2026

Dear Editor and Reviewers,

We would like to thank the editorial team and the reviewers for taking time to review our manuscript and providing detailed comments. The reviewers both recognized the importance of this work and provided many valuable suggestions. We have carefully addressed all comments, and our point-by-point responses are detailed below. All changes to the main text are colored in blue in our submitted revised manuscript.

## Reviewer 1

**Reviewer Comment:** *The novelty is somewhat limited. The idea of fine-tuning diffusion models or flow matching have been explored in molecular generation [1]. The strategy for fine-tuning is simply an adaption from [2].*

**Response:** We thank the reviewer for raising this constructive comment. We agree that the work of Gu et al. [1] is related to ours, but they proposed a method that aligns the target distribution with user-defined preference $\sigma(r^w - r^l)$, where $r^w$ and $r^l$ denote the rewards of a pair of winning and losing ligands, and $\sigma$ denotes the sigmoid function. Such methods require the construction of a fine-tuning dataset with pairwise scored data in advance. Our method optimizes the agent model along with sampling process, alleviating the need of collecting data before training. Nevertheless, we have positioned our work against Gu et al in the Introduction section (line 80-83). Finally, although the continuous fine-tuning strategy is based on Fan et al. [2], we further extended to discrete flows and for the first time applied to property-controllable molecular generation task.

**Reviewer Comment:** *In section 2.3, how do you define the preference of different objectives.*

**Response:** In section 2.3, we defined a Multiple Parameter Optimization (MPO) score function and tested the performance of our reinforcement learning method. In the experiment, two components (logP and $p(\text{active}|\text{molecule})$) of the score function are equally weighted as 1.0 without preference. However, the form of our score function supports incorporating different weighting factors for score components (Eq. 13, line 376), thereby allowing preference to more interested properties.

**Reviewer Comment:** *The experiments are weak. The performance is only conducted between the prior model and the fine-tune model. It is important to compare the fine-tuned model with others state-of-the-art methods and apply the fine-tuning strategy to different prior models.*

**Response:** We appreciate the reviewer for the comment. We would like to clarify that our strategy adapts to any kind of flow matching models. Therefore, by following the reviewer's suggestion, we have evaluated our RL framework combined to another state-of-the-art 3D molecular

generative model, FlowMol3 [3]. The results are reported in Appendix D (from line 680), where our RL framework is used to optimize $p(\text{active}|\text{molecule})$ and the strain energy, the same objective function in Section 2.2. As shown in Figure 7, the agent based on the FlowMol3 model is able to produce more ligands with higher $p(\text{active}|\text{molecule})$ while to maintain the strain energy in acceptable range across all atom numbers (see Table 7). The active molecules generated by the agent reaches 4 times of that generated by the prior model. This demonstrates that our RL framework is transferrable to other flow matching based model architectures.

# Reviewer 2

**Reviewer Comment:** *The experimental evaluation is somewhat limited in scope. The comparisons are primarily between the original prior model and the proposed reinforcement-learning-modified variant of the same model. This makes it difficult to assess the overall impact of the proposed steering technique relative to other modern generative models. Several recently developed 3D molecular generative models report stronger baseline performance than the architecture used here(eg. Megalodon Flow, FlowMol3, etc). Including comparisons with additional models, or at least discussing their expected behavior under the proposed framework, would strengthen the study.*

**Response:** We thank the reviewer for this comment. This concern was also raised by another reviewer, so we report the same answer here. We would like to clarify that our strategy adapts to any kind of flow matching models. Therefore, by following the reviewer's suggestion, we have evaluated our RL framework combined to another state-of-the-art 3D molecular generative model, FlowMol3 [3]. The results are reported in Appendix D (from line 680), where our RL framework is used to optimize $p(\text{active}|\text{molecule})$ and the strain energy, the same objective function in Section 2.2. As shown in Figure 7, the agent based on the FlowMol3 model is able to produce more ligands with higher $p(\text{active}|\text{molecule})$ while to maintain the strain energy in acceptable range across all atom numbers (see Table 7). The active molecules generated by the agent reaches 4 times of that generated by the prior model. This demonstrates that our RL framework is transferrable to other flow matching based model architectures.

**Reviewer Comment:** *Even simple engineered baseline for steering could provide useful context. For example, one could consider resampling the training data based on the reward function(or target property) and retraining the model. Such baselines would help isolate how much of the observed improvement arises specifically from the proposed reinforcement learning approach.*

**Response:** We thank the reviewer for this valuable suggestion, and add this baseline experiment in Appendix C (from line 670). Here, we sampled 64,000 molecules with the prior model, and retained the top 10% highest scored ligands. Those molecules were used to fine-tune the agent model for 1,000 steps with batch size 64. Figure 6 plots the strain energy against $p(\text{active}|\text{molecule})$ of molecules generated by this baseline model, RL-trained model and prior model. RL training promotes the upper bound of $p(\text{active}|\text{molecule})$ and reduce the proportion of high strain energy molecules, which are remained in baseline model generation.

**Reviewer Comment:** *Additional, ablation studies would also be helpful. Particularly, regarding the regularization term used to constrain the agent model toward the prior. Since this term is introduced to mitigate policy collapse and appears central to training stability, evaluating its influence on the final performance would clarify how sensitive the method is to this design choice. It can be justifyible to use it as it is if it is absolute standard of such models. In this case I would suggest change wording on it.*

**Response:** We appreciate the reviewer for this advice. The regularization term $\lambda_r$ has played

an important role in the stability of our algorithm. To further evaluate its influence on the efficacy of our approach, we fine-tuned a series of agents with the objective of decreasing PSA (Section 2.1) under $\lambda_r$ ranging from 0.01 to 0.2. We then use each of the agent models to generate 200 molecules and compare their validity ratio and averaged scores. As indicated in Figure 8, the current chosen $\lambda_r = 0.1$ is a balance between generation validity and the target score optimization. $\lambda_r$ lower than 0.1 would enhance agent's performance on the target property, while face a higher risk of producing less valid molecules. More details please refer to Appendix E (from line 690).

**Reviewer Comment:** *Steering the model appears to introduce trade-offs in structural quality. In several experiments the validity decreases and strain energy increases compared to the prior model (in some error is increasing around 80% when validity errors increases from 12% to 20%). Also from the results it is obvious that this degradation depend on the property itself (some exhibits larger drops). While this behavior is briefly mentioned, the manuscript would benefit from a more detailed discussion of these effects. In particular, it would be helpful to understand whether the degradation can be controlled by adjusting the regularization coefficient, modifying the reward function, or introducing additional structural constraints during training.*

**Response:** We thank the reviewer for pointing out this concern. The drop in validity is something we have thoroughly investigated. We found that in the original SemlaFlow implementation, there was an unstable attention mechanism in Equation 10 of the original paper [4], implemented as

$$\hat{x}_{i,j} = \frac{\tilde{x}_j - \tilde{x}_i}{\|\tilde{x}_j - \tilde{x}_i\| + \epsilon},$$

where $\epsilon$ is a small constant. While this formulation is correct, its gradient with respect to $\tilde{x}_i$ (used when training the agent by backpropagation) is unstable:

$$\nabla_{\tilde{x}_i} \hat{x}_{i,j} = -\frac{I}{\|\tilde{x}_j - \tilde{x}_i\| + \epsilon} + \frac{(\tilde{x}_j - \tilde{x}_i)(\tilde{x}_j - \tilde{x}_i)^T}{\|\tilde{x}_j - \tilde{x}_i\|(\|\tilde{x}_j - \tilde{x}_i\| + \epsilon)^2}.$$

The second term of the gradient becomes numerically unstable because the $\epsilon$ cancels in the first factor of the denominator, which can cause issues when $\tilde{x}_j$ and $\tilde{x}_i$ are very close to each other. We address this by modifying the layer as follows:

$$\hat{x}_{i,j} = \frac{\tilde{x}_j - \tilde{x}_i}{\sqrt{\|\tilde{x}_j - \tilde{x}_i\|^2 + \epsilon^2}}.$$

With this formulation, the gradient remains stable:

$$\nabla_{\tilde{x}_i} \hat{x}_{i,j} = -\frac{I}{\sqrt{\|\tilde{x}_j - \tilde{x}_i\|^2 + \varepsilon^2}} + \frac{(\tilde{x}_j - \tilde{x}_i)(\tilde{x}_j - \tilde{x}_i)^T}{(\|\tilde{x}_j - \tilde{x}_i\|^2 + \varepsilon^2)^{3/2}}.$$

After this modification we have redone all the experiments and as can be observed in Tables 1, 2, 3, 4, the degradation of the validity is strongly mitigated.

**Reviewer Comment:** *The tables report only single average values without any indication of statistical uncertainty. Since the evaluation relies on stochastic sampling of generated molecules, reporting standard deviations or confidence intervals across multiple runs (or subsets) would make the comparisons more informative and scientific. Including reference values from the underlying dataset would also help contextualize some metrics. For example, reporting QED statistics for molecules within the desired property range in the training data would provide a useful baseline for interpreting the generated results.*

**Response:** We appreciate this helpful advice. In response, we have re-run the each experiments of each objective for five times and incorporated the mean and standard deviation from five

repeated runs into all tables. In addition, we have added another row to report the corresponding metrics of GEOM-Drugs dataset that is used for training the prior model.

**Reviewer Comment:** *Some evaluation metrics would benefit from clearer explanation. In particular, the strain energy is reported in kcal/mol per atom. For typical drug-like molecules with tens of atoms, the reported values correspond to very large total strain energies (3 per atom means 150 for 50 atoms molecule, which is like bond breaking energy, while typical conformational energies do not exceed 5 kcal/mol). Clarifying how this metric is calculated and normalized would improve interpretability. It would also be helpful to ensure that the energy model used for evaluation is consistent with the one associated with the dataset or training setup, as inconsistent energy definitions can lead to misleading comparisons (https://doi.org/10.1039/D5DD00206K).*

**Response:**

Thanks the reviewer for giving us the chance to clarify this issue. The reason we reported the strain energy in form normalized by the number of atoms, is to compare fairly across molecules with different sizes. As the reviewer noticed, strain energies of generated molecules after RL training were generally higher than that from the prior model in our preliminary submission. This phenomenon has been greatly improved in the revised manuscript in which the issue of unstable gradient computation has been fixed that all strain energies are evaluated lower than 5 kcal/mol/atom. On the other hand, agent model's performance on strain energy is closely aligned with the prior model. For example, the prior FlowMol3 model is able to generate molecules with globally lower strain energies (0.52 kcal/mol/atom), and the agent model trained from it with our approach reported a relatively lower strain energy as well (0.92 kcal/mol/atom). Therefore, better prior model would lead to better strain energy after RL training.

For generated models like SemlaFlow, such flaw in strain energy usually comes from the imperfection of generated 3D coordinates. A tiny movement from the ideal positions leads to a surge in strain energy computation. If we compare the root mean square deviations (RMSD) between the raw generated molecules and the post-processed ones after energy minimization with MMFF94, the average RMSD is $0.17\mathring{A}$, indicating the small deviation between the generated raw molecules and their relaxed states. Thus in practice, we'll do post-optimization to molecules generated from these models before further application. In addition, including strain energy as one of the components of score function during RL training is another feasible way to alleviate this problem, as we showed in Appendix B.

In this manuscript, we've chosen the MMFF94 force field potential energy to evaluate strain energy. As kindly pointed out by the reviewer, this choice is less precise than GFN2-xTB potential that is used to generate conformers in GEOM-Drugs, which constitutes the training dataset of prior models. However, such error induced by the misaligned potential functions can be neglected when used in RL training. As shown in Table 1, 2, 3 and 4, we evaluated MMFF94 strain energy for 5,000 molecules sampled from GEOM-Drug dataset, the averaged values are all near to zero, showing the inconsistency of energy evaluation is not the driven factor of lower strain energy measurements.

## References


[1] Gu, S. *et al.* Aligning Target-Aware Molecule Diffusion Models with Exact Energy Optimization (2024). 2407.01648.

[2] Fan, J. *et al.* Online Reward-Weighted Fine-Tuning of Flow Matching with Wasserstein Regularization (2025). 2502.06061.

[3] Dunn, I. & Koes, D. R. FlowMol3: Flow matching for 3D de novo small-molecule generation. *Digital Discovery* (2026).

[4] Irwin, R., Tibo, A., Janet, J. P. & Olsson, S. Efficient 3D Molecular Generation with Flow Matching and Scale Optimal Transport (2024). 2406.07266.

# Response to Reviewers: Controllable Molecular Generation with Fine-tuned Flow-Matching Model

Kunyu Wang, Jon Paul Janet, Alessandro Tibo

August 6, 2026

Dear Editor and Reviewers,

We would like to thank the editorial team and the reviewers once again for their patience and valuable feedback. Regarding the remaining concern raised by Reviewer 1, we provide our response below. All changes made to the main text since the previous submission are highlighted in blue in our submitted revised manuscript.

## Reviewer 1

**Reviewer Comment:** *I appreciate the authors' responses, and I am satisfied with most of the clarifications provided. However, I still have a remaining concern regarding my last comment. My point is that the proposed method should be compared with a broader range of property optimization methods. Specifically, to better demonstrate the advantage of the proposed algorithm over related optimization strategies, I suggest using the same pretrained prior model and applying different fine-tuning or optimization methods under a consistent setting. This would allow a more direct and fair comparison of the optimization capability of the proposed approach. Potential baselines to consider include AliDiff [1], DecompDPO [2], DECOMPOPT [3], and MolJO [4].*

**Response:** We thank the reviewer for further this comment. To compare our method with other property optimization strategies, we conducted the same property optimization experiments using our method, AliDiff [1], and DecompDPO [2] (suggested by the reviewer). For a fair comparison, we followed the same online RL training process described in Algorithm 1, replacing only the loss function with that of each baseline method. Compared with the baselines, our method generated more molecules that satisfied the desiderata criteria with much lower variance across 5 independent runs. More experimental details and results are provided in Supplementary Information Section 6. Unfortunately, the other two baseline methods, DECOMPOPT [3] and MolJO [4], are not applicable to our setting. DECOMPOPT [3] relies on iterative refinement of template fragments to optimize the target properties of generated molecules, whereas our pretrained model doesn't require template fragments as priors. MolJO [4] requires the reward function, or a surrogate predictive model of the reward, to be differentiable. As discussed in the Introduction (line 68-73), most optimization objectives in industrial drug development are based on non-quantitative features and are therefore non-differentiable, and many are costly to be approximated with surrogate models. Nevertheless, we have discussed all four methods in the Introduction to better position our method within a broader overview of existing optimization strategies (line 66-68, 82-88).

## References


[1] Gu, S. *et al.* Aligning Target-Aware Molecule Diffusion Models with Exact Energy Optimization (2024). 2407.01648.

[2] Cheng, X., Zhou, X., Yang, Y., Bao, Y. & Gu, Q. Decomposed Direct Preference Optimization for Structure-Based Drug Design (2026). 2407.13981.

[3] Zhou, X. *et al.* DecompOpt: Controllable and Decomposed Diffusion Models for Structure-based Molecular Optimization (2024). 2403.13829.

[4] Qiu, K. *et al.* Empower Structure-Based Molecule Optimization with Gradient Guided Bayesian Flow Networks (2025). 2411.13280.

# Response to Reviewers: Controllable Molecular Generation with Fine-tuned Flow-Matching Model

Kunyu Wang, Jon Paul Janet, Alessandro Tibo

August 16, 2026

Dear Editor and Reviewers,

We would like to thank the editorial team and the reviewers once again for their final remarks and feedbacks. We believe we have now addressed all the requested editorial changes. We provide our detailed response below.

## Editor

**Reviewer Comment:** *We notice that you have deposited your code in a GitHub repository, which we fully support. However, even if you specify the exact commit used in this work, GitHub links are not permanent and can be modified (e.g. by changing the folder name), which would render the link broken and the reproducibility information lost. Therefore, in addition, please make your code citable by obtaining a DOI for the GitHub repository in order to provide a permanent reference to the version of the code used in this study and improve reproducibility. This can be done by linking the repository to Zenodo, following the instructions here: https://guides.github.com/activities/citable-code Alternatively, you can deposit the code in Gigantum or Code Ocean for the same purpose. Please add a reference item for your repository to your list following the format, "authors, title (this paper), repository name, DOI identifier, year." and cite it in in your manuscript text or Code Availability statement.*

**Response:** Thanks! We have created a DOI with Zenodo, added the reference as stated in the guidelines, specifically *Kunyu Wang, Jon Paul Janet, and Alessandro Tibo. Controllable molecular generation with fine-tuned flow-matching model, MolecularAI/flow_matching_rl, 2026a. URL https://doi.org/10.5281/zenodo.21806881.* We have also cited the code in the Code Availability section.

## Assistant Editor

**Reviewer Comment:** *Please add a reference item for your repository to your list following the format, "authors, title (this paper), repository name, DOI identifier, year." and cite it in in your manuscript text or Code Availability statement, alongside the URL.*

**Response:** Thanks! We have created a DOI with Zenodo, added the reference as stated in the guidelines, specifically *Kunyu Wang, Jon Paul Janet, and Alessandro Tibo. Controllable molecular generation with fine-tuned flow-matching model, MolecularAI/flow_matching_rl, 2026a. URL https://doi.org/10.5281/zenodo.21806881.* We have also cited the code in the Code Availability section.

**Reviewer Comment:** *Please rename all figures and tables in the Supplementary Information file as Supplementary Figure/Table XX and update the corresponding citations throughout the article accordingly*

**Response:** All figures and tables are renamed to Supplementary Figures and Tables, respectively in both captions and text in the Supplementary Information as requested.

**Reviewer Comment:** *Please provide the description for "Supplementary data 1" in the "Description of additional supplementary word file" uploaded along with the other files*

**Response:** Thanks! We have provided the following description in "Description of additional supplementary word file" for "Supplementary data 1": Raw data supporting the reinforcement learning-based molecular generation and optimization results reported in the manuscript. The data are organized into three folders containing generated molecules across different tasks and methods in SDF format, along with CSV files containing the molecular rewards at each epoch for the experiments described in Section 6 of the Supplementary Information. This data can be used to regenerate all the figures in the paper using the provided Jupyter notebook.
