# Supplementary material for: Controllable molecular generation with fine-tuned flow-matching model
Source: Commun Chem. 2026 Sep 5;9:291. doi: 10.1038/s42004-026-02188-z (PMC13546285; doi:10.1038/s42004-026-02188-z)
Supplement: Supplementary file 3 [file 42004_2026_2188_MOESM3_ESM.pdf]

Supplementary data 1:

Raw data supporting the reinforcement learning-based molecular generation and optimization results reported in the manuscript. The data are organized into three folders containing generated molecules across different tasks and methods in SDF format, along with CSV files containing the molecular rewards at each epoch in experiments of Supplementary Information Section 6. This data can be used to regenerate all the figures in the paper using the provided Jupyter notebook.
